# The Feeling Is Mutual: Clarity of Haptics-Mediated Social Perception Is Not Associated With the Recognition of the Other, Only With Recognition of Each Other

**DOI:** 10.3389/fnhum.2020.560567

**Published:** 2020-09-04

**Authors:** Tom Froese, Leonardo Zapata-Fonseca, Iwin Leenen, Ruben Fossion

**Affiliations:** ^1^Embodied Cognitive Science Unit, Okinawa Institute of Science and Technology Graduate University, Okinawa, Japan; ^2^Plan of Combined Studies in Medicine (PECEM), Faculty of Medicine, National Autonomous University of Mexico, Mexico City, Mexico; ^3^Center for the Sciences of Complexity (C3), National Autonomous University of Mexico, Mexico City, Mexico; ^4^Section Phenomenological Psychopathology and Psychotherapy, Department of General Psychiatry, Center of Psychosocial Medicine, University of Heidelberg, Heidelberg, Germany; ^5^Faculty of Psychology, National Autonomous University of Mexico, Mexico City, Mexico; ^6^Institute of Nuclear Sciences, National Autonomous University of Mexico, Mexico City, Mexico

**Keywords:** embodied cognition, social cognition, enactive approach, virtual reality, agency detection, perceptual awareness scale

## Abstract

The enactive theory of perception hypothesizes that perceptual access to objects depends on the mastery of sensorimotor contingencies, that is, on the know-how of the regular ways in which changes in sensations depend on changes in movements. This hypothesis can be extended into the social domain: perception of other minds is constituted by mastery of self-other contingencies, that is, by the know-how of the regular ways in which changes in others’ movements depend on changes in one’s movements. We investigated this proposal using the perceptual crossing paradigm, in which pairs of players are required to locate each other in an invisible one-dimensional virtual space by using a minimal haptic interface. We recorded and analyzed the real-time embodied social interaction of 10 pairs of adult participants. The results reveal a process of implicit perceptual learning: on average, clarity of perceiving the other’s presence increased over trials and then stabilized. However, a clearer perception of the other was not associated with correctness of recognition as such, but with both players correctly recognizing each other. Furthermore, the moments of correct mutual recognition tended to happen within seconds. The fact that changes in social experience can only be explained by the successful performance at the level of the dyad, and that this veridical mutual perception tends toward synchronization, lead us to hypothesize that integration of neural activity across both players played a role.

## Introduction

Imagine you are going on a romantic date at the cinema. Inside the movie theater you sit down next to your date, but it is so dark that you cannot see each other, leaving you uncertain about their presence. At some point during the movie you feel your date’s hand touching your hand, and you start holding hands. Your experience of watching the movie is transformed, as it takes on a more distinctively social quality: “we” are sharing this experience with each other. How can such embodied social interaction have this profound effect on an individual’s perceptual experience?

This study aimed to investigate how a person’s real-time tactile interaction with other people can make an irreducible difference to how that person experiences their self, others, and the world, an effect which has been referred to as “genuine intersubjectivity” (Froese, 2018; Froese and Krueger, forthcoming). It builds on the key role of interpersonal contingencies that emerge from the coupling of human bodies Dumas et al., 2014). This aim and basis stand in sharp contrast to what has been characterized as the “methodological individualism” of traditional cognitive science (Boden, 2006), which in its more extreme formulations has even taken an isolated brain as the in-principle sufficient basis of all social experience (Searle, 1990). Nevertheless, the notion of genuine intersubjectivity is consistent with a small but growing number of psychological and neuroscientific experiments as well as agent-based simulation studies, which point to the constitutive role of social interaction for social cognition (e.g., De Jaegher et al., 2010; Schilbach et al., 2013; Candadai et al., 2019).

**Figure 1:**
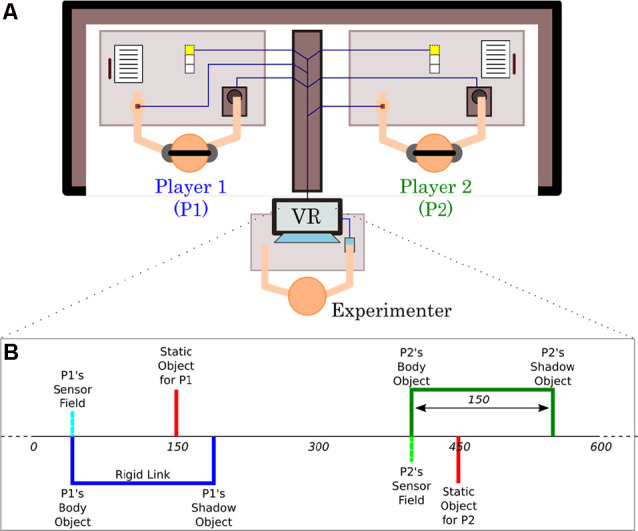
Experimental setup of the perceptual crossing paradigm. **(A)** The physical setup. The two participants can only engage with each other *via* a haptic human-computer interface that reduces their scope for bodily interaction to a minimum of horizontal left to right movement and tactile sensation. Each player’s interface consists of two parts: (1) a trackball that controls the displacement of their “avatar” in an invisible 1D virtual environment; and (2) a hand-held haptic feedback device that vibrates at a constant frequency for as long as the avatar overlaps another virtual object and remains off otherwise. **(B)** The virtual setup. Players are embodied as minimal avatars on an invisible line that wraps around after 600 units of space. Each avatar consists of a binary contact sensor and a body object. Unbeknownst to the players a “shadow” object is attached to each avatar body at a fixed distance of 150 units. There are also two static objects, one for each player. All objects are four units long and can therefore only be distinguished interactively in terms of their different affordances for engagement.

A particularly promising methodology for studying the effects of real-time embodied social interaction is the so-called “perceptual crossing” paradigm, which was originally proposed by Lenay and colleagues (Lenay et al., 2006; Auvray et al., 2009). Pairs of participants are connected to an invisible 1D virtual space using a minimal haptic computer interface, and their task is to locate the other person’s avatar based on the patterns of interaction while avoiding distractor objects (see Figure 1). This paradigm has inspired several experimental variations and different applications (for a review, see Auvray and Rohde, 2012; Deschamps et al., 2016; Zapata-Fonseca et al., 2018; Barone et al., 2020). Since the first studies, there have been discussions of participants’ anecdotal reports of their social encounters (Lenay, 2010; Auvray and Rohde, 2012). However, to investigate genuine intersubjectivity more experimentally, such studies must also include an explicit assessment of participants’ lived experience.

An important step in this direction was provided by a variation of the perceptual crossing paradigm by Froese et al. (2014a), in which participants were asked to rate the clarity of their perception of the other’s presence. They found that a participant’s perceptual clarity was a joint achievement: it was not associated with a participant’s correct identification of the other *per se*, but rather with bidirectional interactions that permitted both participants to successfully recognize each other. This conclusion was further supported by subsequent explorations of the same dataset (Froese et al., 2014b; Zapata-Fonseca et al., 2016; Kojima et al., 2017). This was the first empirical proof of the concept of genuine intersubjectivity.

However, given that this was an isolated experiment, it remained to be seen whether it could be replicated. This has now been accomplished by Hermans et al. (2020), as well as by the present study. Hermans et al. (2020) implemented a shorter variation of Froese et al.’s (2014a) perceptual crossing paradigm as part of a longitudinal population study of adolescents. Importantly, despite this reduction in overall interaction time, as well as differences in the target population and in the assessment of participant’s experience, they also found that subjective experience was highest specifically in trials when both participants were jointly correct in detecting each other’s presence.

### Hypotheses

In the present study, we replicated Froese et al.’s experimental setup and tested several hypotheses related to genuine intersubjectivity. This concept is best approached by drawing on the theoretical resources of the phenomenological tradition in philosophy, especially its work on the phenomenology of direct social perception (Gallagher, 2008; Krueger, 2012), and of the enactive approach to cognitive science, especially the enactive theory of perception (Noë, 2012) and its extension to social perception (De Jaegher, 2009; McGann and De Jaegher, 2009). In essence, this theory holds that object perception consists in the skillful regulation of sensorimotor interaction, which involves knowing how sensations of the object would change concerning possible movements of one’s body, and which provides direct access to the object of perception. An individual’s mastery of these dependencies of sensations on bodily movements, that is, of so-called sensorimotor contingencies, entails a better perceptual grasp of the perceived object.

The same idea of sensorimotor contingencies can also be applied to the perception of another person. It is known that the motor system is involved in social cognition, which is often interpreted as simulation or mirroring (Rizzolatti and Sinigaglia, 2016), but can also be considered as enactive perception in a social context (Gallagher, 2009; but see Gallese, 2017). More specifically, in line with sensorimotor theory (O’Regan et al., 2005), it has been proposed that social perception consists in the skillful co-regulation of social interaction (De Jaegher et al., 2010; Froese and Di Paolo, 2011), which involves both participants knowing how sensations caused by the other’s bodily movements would change concerning their possible bodily movements, and the mastery of these “self-other contingencies” thereby provides access to the other person (McGann and De Jaegher, 2009).

According to this enactive theory, there is a crucial difference between social perception and object perception in terms of their respective conditions of successful perceptual access: both depend on skillful regulation of interaction, but in the case of perceiving another person as another person this access additionally depends on a complementary skillful response by the other person. If the other does not respond appropriately, the perceptual situation would be more akin to that of object perception, for example of perceiving the other’s physical body. This theory, therefore, predicts that the basis of social perception goes beyond the individual perceiver to include another perceiver. However, it is not yet entirely clear what this inter-personal basis of social perception means for the perceiver’s experience. At least two possibilities present themselves, which we will refer to as weak and strong forms of genuine intersubjectivity, respectively:

(1) *Weak genuine intersubjectivity*: This possibility accepts that the basis of social perception can be distributed across two persons, such that one person’s perception of the other person is partly constituted by their ongoing social interaction. However, it remains conservative about the boundaries of consciousness, because it still maintains that each person’s experience remains the property of only that individual. This implies that there are two independent, non-overlapping experiences; each person’s social perception can be shaped by the other’s movements, but without ever constituting a single, jointly shared moment of experience.
(2) *Strong genuine intersubjectivity*: This possibility also accepts that the basis of social perception can be distributed across two persons, but it is more liberal about the boundaries of consciousness. It rejects the claim that two interacting persons must always have two independent experiences and instead accepts the possibility that an interactively extended basis can also give rise to one jointly unfolding experience, for instance of mutually perceiving each other. This implies that this single, jointly shared experience is better characterized as a moment of co-presence that is grasped from each perceiver’s specific point of view.

The possibility of weak genuine intersubjectivity implies that two people in interaction can have their experience shaped by that ongoing interaction at different times. The possibility of strong genuine intersubjectivity, on the other hand, implies tighter inter-personal integration, which fits well with growing evidence that there is a synchronization of neural activity across brains during social interaction, including in the faster frequency bands, and that this is the basis for inter-personal neuronal integration that has implications for different aspects of social cognition (Valencia and Froese, 2020).

More specifically, Froese (2018) has suggested extending Varela’s (1999) neuro-phenomenological analysis of present-time consciousness to the social domain. The idea that genuine intersubjectivity requires interpersonal integration at the most fundamental level of temporality resonates with research in the phenomenology of consciousness (Rodemeyer, 2010), and complements it with a scientific methodology. Varela highlights that the conscious moment of “now” is not an instant but has a duration of 1–3 s. He argues that this duration results from the amount of time it takes for neural activity in an individual’s brain to become transiently integrated *via* long-range synchronization.

Accordingly, an attractive hypothesis of how two people could experience that “we” are sharing one and the same “now,” is that their co-regulated social interaction caused their neural activity to become synchronized. We did not directly investigate neural activity in the current experiment, but if this hypothesis is on the right track, then we would expect the time scale of 3 s to be relevant for mutual veridical perception.

We set out to investigate this theory of social perception and the possibilities of weak and strong genuine intersubjectivity in terms of the following hypotheses:

(1) We hypothesized that participants’ capacity to correctly recognize the other will increase over trials, as they learn to redeploy their existing skill of embodied social interaction *via* the haptic computer interface.
(2) We hypothesized that clarity of the other’s perceived presence will increase over trials, as perceptual learning will improve access to the other person.
(3) We hypothesized that increased clarity of the other’s presence will not be explained by the correctness of an individual perceiver’s recognition of the other person, but by the correctness of both perceivers’ recognition of each other, as the shift from object perception to social perception involves a shift from regulation to co-regulation that offers a shared opportunity of recognition to both participants.
(4) We hypothesized that the moments of recognition in trials where both participants correctly recognize each other will tend to be synchronized, specifically in the time scale of 3 s, as a reflection of the shift from individual action to joint action.
(5) We hypothesized that the synchronization of moments of recognition will correlate with the clarity of the perception of the other person, specifically in the time scale of 3 s, as a reflection of the shift from two individual experiences to one intersubjectively shared experience.

Hypotheses 1 and 2 are intended to demonstrate, in behavioral and experiential terms respectively, that an embodied skill of perceiving others is being (re-)acquired throughout the experiment. Hypothesis 3 is aimed at supporting the concept of weak genuine intersubjectivity, i.e., the idea that co-regulated interpersonal interaction makes an irreducible difference to individual experience. Hypothesis 4 and 5 are aimed at supporting the concept of strong genuine intersubjectivity, i.e., that there is a single joint action giving rise to one shared moment of veridical mutual recognition that “we” are now experiencing, based on the assumption that this fusion of individual streams of experiencing will require integration of neural activity across both participants in the time scale of seconds.

## Materials and Methods

We employed a version of the perceptual crossing experiment that was designed to capture subjective reports of participants’ perceptual awareness of the other’s presence to investigate its sensorimotor basis (Froese et al., 2014a,b; Kojima et al., 2017).

### Experimental Equipment

In the perceptual crossing experiment, two participants are seated at separate desks such that they cannot perceive each other visually; they also wear noise-canceling headphones to prevent mutual auditory perception (Figure 1A). Each participant can establish contact with her partner only *via* a simple human-computer interface consisting of two components: (1) a trackball for making horizontal movements; and (2) a hand-located vibration motor that is either on or off. The trackball is operated with the dominant hand and it controls the motions of an avatar located in an invisible circular 1D virtual space (Figure 1B). The motor vibrates as long as the avatar overlaps with another virtual object in this space. Each participant can encounter three objects: (i) the other’s avatar; (ii) a moving object that “shadows” the other’s avatar by following the same trajectory at a distance; and (iii) a static object. Regardless of the object type, the vibratory feedback is only on (and off otherwise).

### Experimental Procedure

Participants were told to work as a team and were asked to come up with a team name, with which they would be ranked against other teams participating in the study. They were instructed to navigate through the invisible shared space to find each other. They were asked to signal with a click (only once per trial) when they became aware of interacting with the other player; for each click correctly identifying their partner the team would gain 1 point, for each wrong click they would lose 1 point. However, no feedback about the correctness of clicks was provided during the experiment. Participants were first individually familiarized with the human-computer interface. Then each pair was tested for 20 trials, consisting of 60 s each (due to errors the last five trials and the last trial of teams 1 and 6, respectively, were not recorded). This is the first time the perceptual crossing experiment has been run for 20 trials; Froese et al. (2014a) had employed 15 trials, whereas Hermans et al. (2020) used only six trials. The aim of using 20 trials was to see if there would be a change in results if participants have more time to interact.

After each trial, the experience of the players was evaluated through questionnaires based on a version of the Perceptual Awareness Scale (PAS), which in its original formulation was used for visual perception (Ramsøy and Overgaard, 2004), but was adapted by Froese et al. (2014a) for social perception. After each trial in which a participant clicked, his or her awareness of the other’s presence at the moment of the click was assessed with values from one to four corresponding to “no experience,” “ambiguous experience,” “almost clear experience,” and “clear experience,” respectively.

The target of each click was categorized as one of the three objects or as unknown. Target assignment was first done automatically based on calculated distances at the time of the click, following (Froese et al., 2014a). This was followed by trial-by-trial visual inspection of plots of the movement trajectories by TF and LZF (see Supplementary Trial Data). Any discrepancies in the target assignment were resolved by TF and LZF in discussion with IL.

### Participants

Twenty adults took part in the perceptual crossing experiment, ages ranged between 18 and 47 years old (median of age = 28), and there were 6 women and 14 men (for details, see Appendix of the Supplementary Statistical Information). Participants were recruited from acquaintances at UNAM in Mexico City. Only healthy volunteers were considered; individuals with neurological, psychiatric, or movement disorders (clinically diagnosed) were excluded. Participation was voluntary and all participants gave their informed consent. Ten teams were created as pairs of volunteers became available. Three teams were composed of strangers, while participants in other teams had some history of interaction from before and some were friends. Some participants were familiar with perceptual crossing from the literature, but none had previous experience of participating in such experiments. Data collection took place between April and May 2018.

### Statistical Analyses

**Figure 2:**
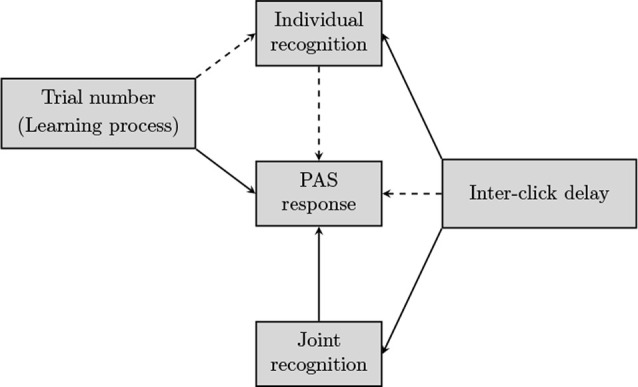
Relations among observed variables at the level of the team (joint recognition, inter-click delay), trial (trial number), and individual participant [individual recognition, Perceptual Awareness Scale (PAS) response], as modeled in the statistical analysis. Dotted arrows indicate that for those relations no direct effect was found.

We specified a statistical model that allowed us to simultaneously examine the relations among the following variables: (a) individual success and (b) joint success in recognizing the other player, (c) the PAS-responses, (d) the inter-click delay (dichotomized, with a value of 1 in case that both players clicked within the same timeframe of 3 s, and 0 otherwise) and (e) the trial number, by which we modeled a learning process for individual successes and PAS-responses across trials. The effects were specified as depicted in Figure 2, by including (binary or ordinal) probit regression submodels for the endogenous variables (individual success, joint success, and PAS-responses); the learning process was modeled through piece-wise regression, with first a learning stage allowing improvement, and subsequently, a consolidation stage where the individual is assumed to operate at the same level. The model is hierarchical (i.e., it includes random effects) as it takes into account the nested structure of the data (with both individuals responding to each trial, and 20 trials for each team) and was fitted using a fully Bayesian approach.

All data can be found in the Supplementary Data Table, while further details of the analysis (together with a complete description of the results) can be found in the Supplementary Statistical Information.

## Results

In total, we recorded 194 trials, for a possibility of 194 × 2 = 388 clicks and PAS-responses. A click was produced in 311 of these 388 cases (i.e., 80%). Of these 311 clicks, 225 (72%) were correct clicks, responding to the other’s avatar. In 79 (41%) of the 194 trials, players produced jointly correct clicks. In 48 (25%) of the 194 trials, both players clicked but either or both gave an incorrect click. There were 307 PAS-responses (four clicks did not receive a PAS-response).

Overall, the frequencies for the four PAS-response categories, ordered from “no experience” to “clear experience,” are 20 (6% out of 307), 91 (30%), 100 (33%), and 96 (31%), respectively. For the 85 PAS-responses associated with an *incorrect* click, the frequencies are 6 (7%), 31 (37%), 34 (40%), 14 (16%), respectively. Responses conditional upon a (65) *correct* click in non-jointly successful trials were: 6 (9%), 30 (46%), 16 (25%), and 13 (20%), while responses conditional upon a (157) *correct* click in *jointly* successful trials were: 8 (5%), 30 (19%), 50 (32%), and 69 (44%).

We summarize here the main findings from the detailed statistical analysis included as Supplementary Statistical Information, focusing on the hypotheses listed in the introduction:

(1) We used a piecewise regression model for the individual learning process of correctly recognizing the other player, with a learning stage and a consolidation stage. On average, learning takes place between trial 1 and trial 3.2 [95%-high posterior density interval = (1.7, 4.9)]; however, there are large differences, with individuals who apparently do not enter in a learning process and others for whom learning takes place until half the experiment.
(2) Concerning the learning process on experiential clarity, the breakpoint that separates the learning and consolidation stage is situated, on average, at trial 5.1 [95%-HPDI = (1.1, 9.1)], but again with a relatively large variance^1^.
(3) The results do not show evidence of an effect of individual success in recognizing the other on the PAS-responses [with an estimated effect on the probit scale of β = −0.16; 95%-HPDI = (−0.55, 0.20)], whereas joint success does lead to higher PAS-values [*β* = 0.69; 95%-HPDI = (0.31, 1.06)].
(4) A short inter-click delay, of less than 3 s, goes with a higher probability of individual success [with an estimated effect on the probit scale of *β* = 0.70, 95%-HPDI = (0.16, 1.29) as well as a higher probability of joint success in recognizing the other [*β* = 1.01, 95%-HPDI = (0.40, 1.59)].
(5) There is no clear evidence of a direct association between short inter-click delays and PAS-responses [*β* = 0.14, 95%-HPDI = (−0.28, 0.54)]. Note, however, that there is an indirect effect given that short inter-click delays are associated with higher probabilities of joint successful recognition (see the previous point), which in turn leads to higher PAS-responses (see Point 3).

## Discussion

These results support genuine intersubjectivity, although evidence for strong genuine intersubjectivity remains indirect.

First, on average, participants’ perceptual experience of the other’s presence became clearer during the experiment. This change in experience tended to stabilize within six trials, and our study thereby supports Hermans et al.’s (2020) decision to run a shorter experiment of six trials. However, it is noteworthy that we did not find compelling evidence that, on average, participants improved their capacity to click correctly. Some improved quickly, others were slow learners, and some never improved even though we had extended the number of trials to 20 trials. Future work could investigate the reasons for this diversity in learning outcomes; presumably, the recognition task is facilitated if participants have a history of close interaction, and there may also be an influence of individuals’ age and sex, which are factors that we did not take into consideration in the current analysis. Yet the fact that clarity of social presence increased, and did so independently of individuals’ success at objectively recognizing the other, also suggests that an explanation of this phenomenological change should look beyond individuals.

Second, indeed, we found compelling evidence for genuine intersubjectivity: an individual’s increased perceptual clarity of the other’s presence could only be explained by taking into account task performance at the level of the dyad, and not at the level of the individuals. In other words, an individual’s correct recognition of the other was necessary but not sufficient to explain the changed quality of perceptual experience; the other’s involvement, as measured in terms of their correct recognition of the self, was also necessary.

It is reassuring that we managed to replicate this dependence of perceptual experience on the interaction process using a comprehensive statistical model. This key finding is in tension with the traditional view of perception as a brain-based process of furnishing mental representations inside of the individual. Instead, it fits better with the enactive view that the basis of perceptual experience extends into sensorimotor interaction, which in the case of social perception also includes a relationship with another subject. Future work could try to uncover these distinctive movement patterns. Based on past analyses, we can expect increases in interdependence as measured by, for example, turn-taking, cross-correlations, and transfer entropy in sensorimotor dynamics (Kojima et al., 2017).

**Figure 3:**
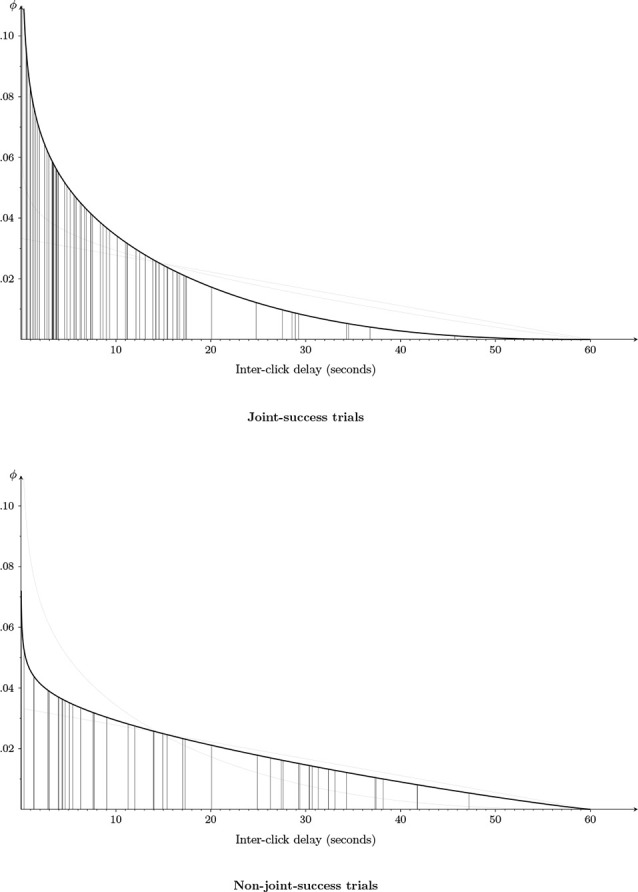
Estimated distribution of inter-click delays for joint-success trials and non-joint-success trials (i.e., where either one or both players produced a click that did not correctly identify the other). The null hypothesis (based on independent uniform distributions of both players’ clicks) is represented by a thin gray line. We graphed the estimated density function separately for both conditions: joint-success trials (upper figure) and non-joint-success trials (lower figure). To allow a better comparison, we show (by a thin gray curve) the estimated density function of the other condition in both figures. Furthermore, the vertical lines connecting the density with the abscissa represent the observed values (79 inter-click intervals for joint success; 48 for non-joint success). Shorter inter-click intervals are more likely than longer intervals. Importantly, comparing joint-success to non-joint-success trials, inter-click intervals tend to be even shorter, with for example the estimated probability that an interval is below 3 s being equal to 0.25 and 0.14, respectively.

**Figure 4:**
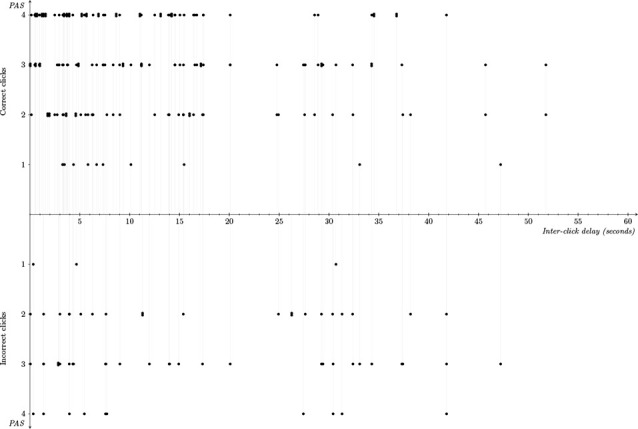
Graphical representation of the relationship between individuals’ responses. Responses were associated with the PAS, individuals’ correctly recognizing the other player (i.e., correct or incorrect click), and the dyads’ inter-click delay in trials where both members of the dyad produced a click. The thin vertical gray lines connect both responses in a dyad that correspond to the same trial and projects the corresponding points to the horizontal axis to show the corresponding inter-click delay.

Third, we found indications of the possibility of interpersonally extended experience of veridical mutual recognition (strong genuine intersubjectivity). As revealed by Figure 3, comparing jointly to non-jointly correct clicks, inter-click intervals tended to be shorter, with the estimated probability that an interval is below 3 s being equal to 0.25 and 0.14, respectively. Also, there was an indirect effect of inter-click intervals within 3 s on increased clarity of social presence, given that those intervals are associated with a higher probability of joint success, which is associated with higher PAS ratings. This tendency of jointly correct 3-s inter-click intervals to be associated with higher PAS ratings can also be seen in Figure 4; future studies with larger sample sizes may still uncover a direct effect.

On the other hand, perhaps we did not develop our hypotheses carefully enough. In contrast to the representational approach to perception, the enactive approach has emphasized that perceptual experience is constituted by certain kinds of organism-environment interaction; hence, the experience is not an entity that is somehow separate from that interaction. Or, more strongly, the perceptual experience is identical with the sensorimotor interaction (Myin and Zahnoun, 2018), or at least not something added to the process (Froese and Taguchi, 2019). Accordingly, the fact that the effect on the clarity of social perception is specifically mediated by jointly correct recognition is consistent with another intriguing possibility: the social quality of experience is constituted by their social interaction. This would also account for the puzzling finding that the residual correlation between PAS-responses of both individuals in a dyad is close to zero, which implies that the other effects included in the model (the learning process, individual and joint success in recognizing the other, and the inter-click delay) may fully account for this correlation.

Overall, this is a promising line of investigation for future work, and it should be possible to further clarify the basis and extent of genuine intersubjectivity by increasing the sample size and by applying a hyperscanning approach to measure neural activity of both participants.

## Data Availability Statement

All datasets presented in this study are included in the article/Supplementary Material.

## Ethics Statement

The studies involving human participants were reviewed and approved by Faculty of Medicine, National Autonomous University of Mexico. The patients/participants provided their written informed consent to participate in this study.

## Author Contributions

TF and LZ-F conceived the idea for this study and designed the experiment. LZ-F and RF carried out the experiment. TF and IL analyzed the data. TF and LZ-F interpreted the results. TF drafted the manuscript. All authors contributed to the article and approved the submitted version.

## Conflict of Interest

The authors declare that the research was conducted in the absence of any commercial or financial relationships that could be construed as a potential conflict of interest.

## Footnotes

^1^As explained in the Supplementary Statistical Information (and due to the small sample size), this and the previous result must be interpreted with caution, as the associated parameter estimates have wide credibility intervals.
