# Supplementary material for: The Feeling Is Mutual: Clarity of Haptics-Mediated Social Perception Is Not Associated With the Recognition of the Other, Only With Recognition of Each Other
Source: Front Hum Neurosci. 2020 Sep 4;14:560567. doi: 10.3389/fnhum.2020.560567 (PMC7500513; doi:10.3389/fnhum.2020.560567)
Supplement: Supplementary file 1 [file Table_1.DOCX]

Supplementary Trial Data

# Supplementary Data

The targets of participants’ clicks were calculated by the procedure introduced by Froese et al. (2014). Subsequently, each trial was plotted (see below) and the automatic target assignments were confirmed by visual inspection of every trial by two authors (TF and LZF), taking into consideration a number of factors familiar from previous perceptual crossing experiments, including spatial proximity between avatars at time of click, amount of preceding perceptual crossing, and occurrence of double stimulation by passing avatar and another nearby object, and the occurrence of passive touch. In situations in which the other’s avatar was present along with the static object, preference was given to the avatar. This choice is justified because previous work has revealed that participants are highly sensitive to sensor activations that were not caused by their own movement (Kojima, Froese, Oka, Iizuka, & Ikegami, 2017). The final assignments were confirmed with a third author (IL).

We also collected additional data that was not included in the current analysis. Following Hermans et al. (in press), after each trial we also measured participants’ sense of the other’s willingness to collaborate. Participants were asked to rate how collaborative the other participant was in performing the task. This collaborativeness scale takes values from 1 to 4 corresponding to “not collaborative”, “somewhat collaborative”, “quite collaborative”, and “fully collaborative”, respectively. The results of the collaborativeness scale could be the target of future analyses.

Before the start of the experiment each of the two participants was also fitted with an Empatica E4 wristband and with a Zephyr BioHarness to record physiological activity. The physiological data was dropped from this analysis because it was very noisy, and we could not rule out that there were technical problems with these recordings.

# Supplementary Figures

Each participant’s rating of their sense of the other’s overall collaborativeness (Col.) is given below each trial. If a participant clicked during the trial, then the target (AV: Avatar object, SH: Shadow object, ST: Static object, UN: Unknown object) and the Perceptual Awareness Scale (PAS) rating of their experience of the other’s presence at the moment of the click are also provided.

Although the PAS ratings are supposed to be associated with clicks only, some participants seemed to have misunderstood this part of the instructions because they reported PAS ratings whether they clicked or not. Here we ignore those other PAS ratings and only report ratings actually associated with clicks, which were the basis for our analysis. Missing values in the questionnaires are indicated with a ‘?’.

***Panel 1: Team 1 (“Belgas”)***


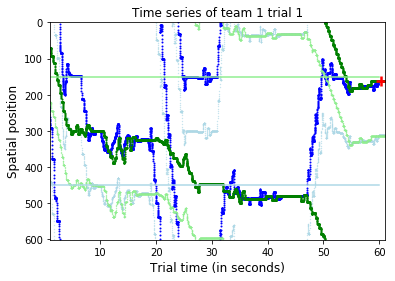

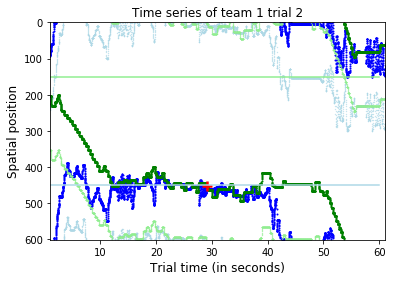

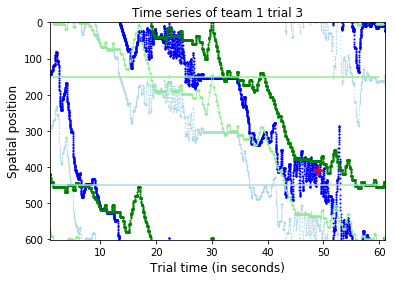


P1’s Col.: 1; Click: AV; PAS: 2 P1’s Col.: 2; Click: AV; PAS: 2 P1’s Col.: 2; Click: AV; PAS: 2

P2’s Col.: 1 P2’s Col.: 1 P2’s Col.: 1


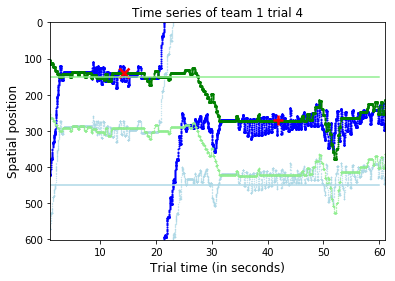

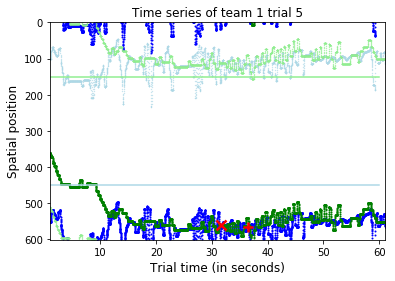

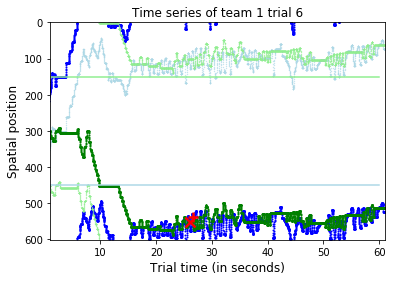


P1’s Col.: 3; Click: AV; PAS: 3 P1’s Col.: 3; Click: AV; PAS: 3 P1’s Col.: 3; Click: AV; PAS: 3

P2’s Col.: 2; Click: AV; PAS: 2 P2’s Col.: 3; Click: AV; PAS: 3 P2’s Col.: 3; Click: AV; PAS: 3


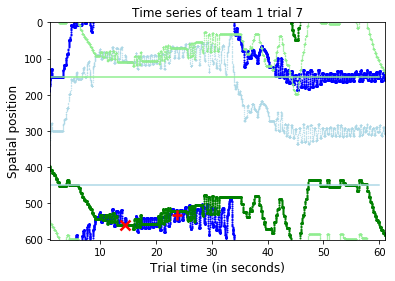

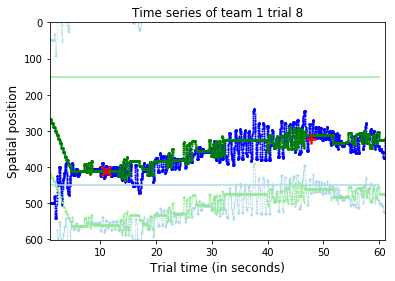

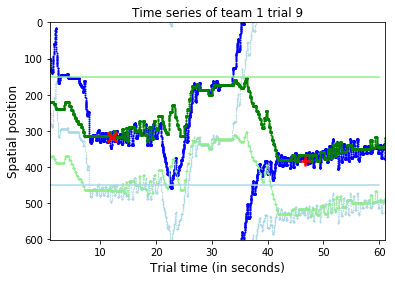


P1’s Col.: 3; Click: AV; PAS: 3 P1’s Col.: 3; Click: AV; PAS: 4 P1’s Col.: 3; Click: AV; PAS: 4

P2’s Col.: 3; Click: AV; PAS: 3 P2’s Col.: 4; Click: AV; PAS: 4 P2’s Col.: 4; Click: AV; PAS: 4


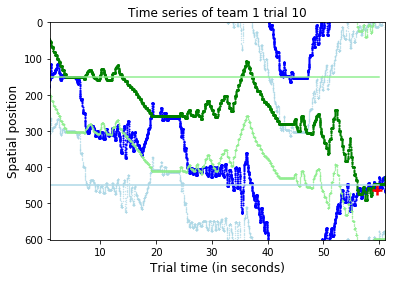

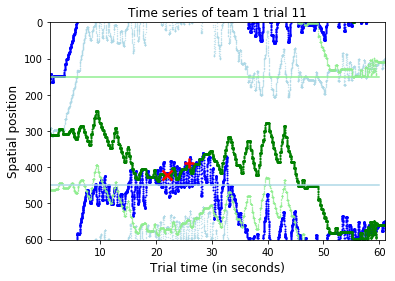

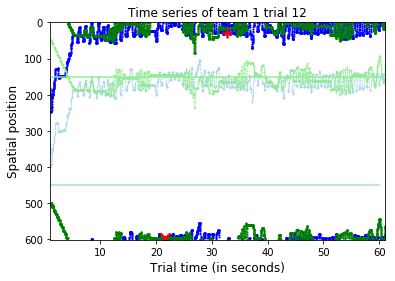


P1’s Col.: 2; Click: AV; PAS: 2 P1’s Col.: 3; Click: AV; PAS: 4 P1’s Col.: 3; Click: AV; PAS: 4

P2’s Col.: 2 P2’s Col.: 4; Click: AV; PAS: 4 P2’s Col.: 4; Click: AV; PAS: 4


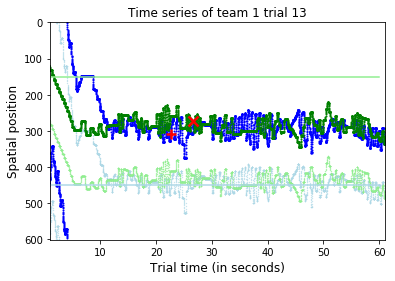

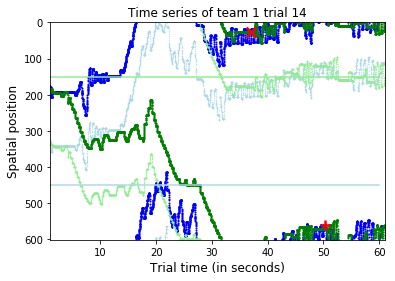

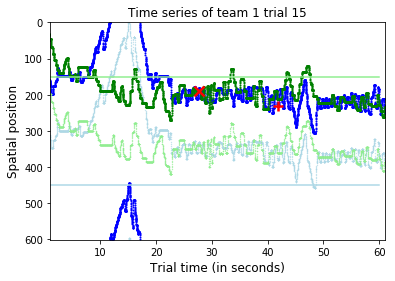


P1’s Col.: 3; Click: AV; PAS: 4 P1’s Col.: 3; Click: AV; PAS: 4 P1’s Col.: 3; Click: AV; PAS: 4

P2’s Col.: 4; Click: AV; PAS: 4 P2’s Col.: 4; Click: AV; PAS: 4 P2’s Col.: 4; Click: AV; PAS: 4

***Panel 2: Team 2 (“Chicos”)***


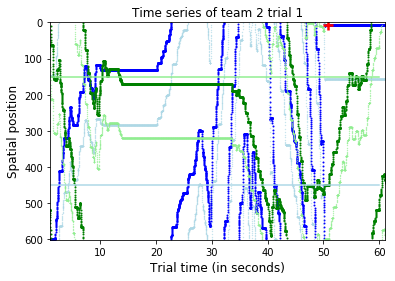

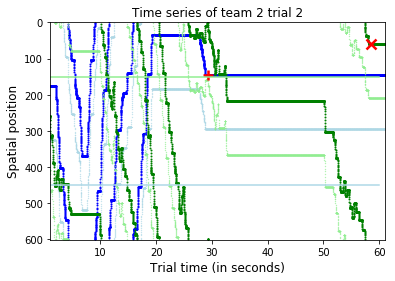

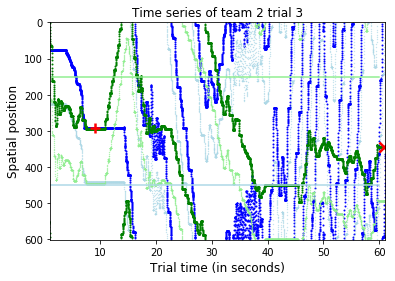


P1’s Col.: 2; Click: SH; PAS: 1 P1’s Col.: 2; Click: ST; PAS: 2 P1’s Col.: 2; Click: AV; PAS: 3

P2’s Col.: 1 P2’s Col.: 2; Click: UN; PAS: 3 P2’s Col.: 2; Click: AV; PAS: 2


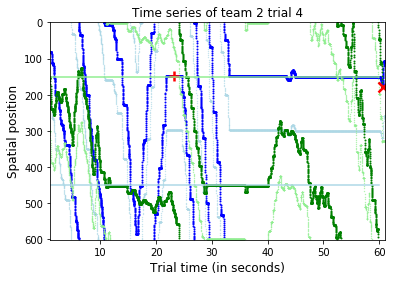

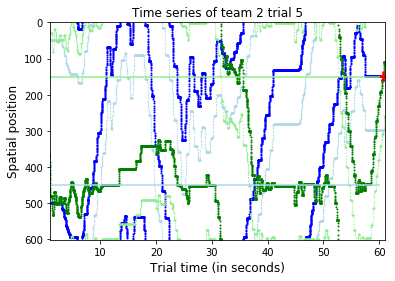

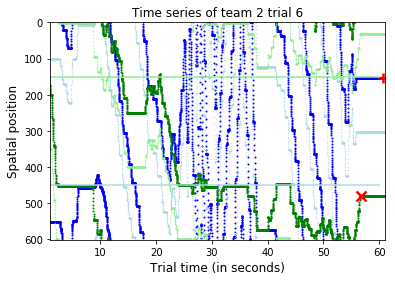


P1’s Col.: 3; Click: ST; PAS: 3 P1’s Col.: 2; Click: ST; PAS: 2 P1’s Col.: 3; Click: ST; PAS: 3

P2’s Col.: 2; Click: AV; PAS: 2 P2’s Col.: 1 P2’s Col.: 2; Click: UN; PAS: 2


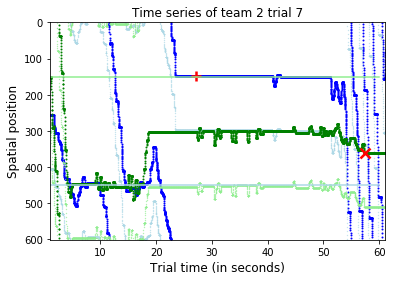

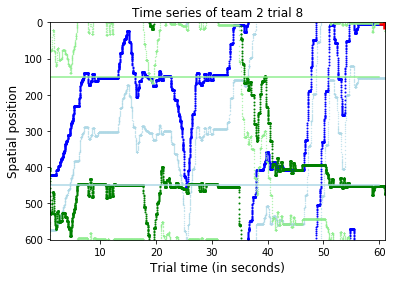

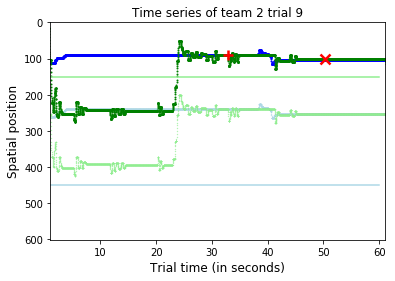


P1’s Col.: 4; Click: ST; PAS: 4 P1’s Col.: 2; Click: SH; PAS: 3 P1’s Col.: 3; Click: AV; PAS: 4

P2’s Col.: 2; Click: SH; PAS: 3 P2’s Col.: 2 P2’s Col.: 2; Click: AV; PAS: 3


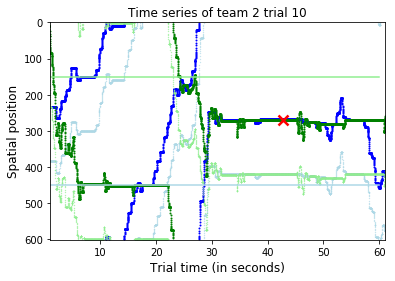

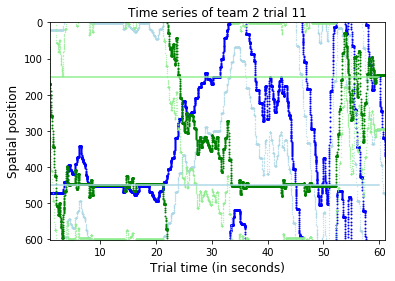

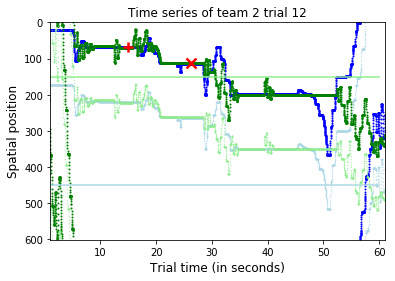


P1’s Col.: 3 P1’s Col.: 3 P1’s Col.: 4; Click: AV; PAS: 3

P2’s Col.: 1; Click: AV; PAS:1 P2’s Col.: 2 P2’s Col.: 2; Click: AV; PAS: 3


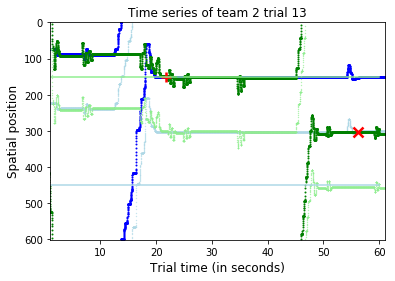

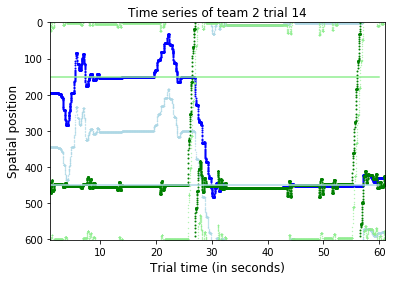

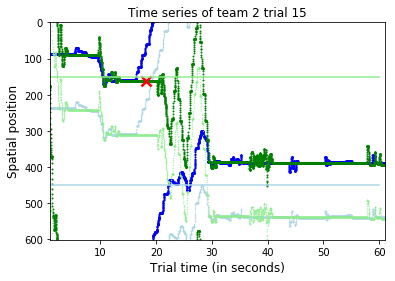


P1’s Col.: 4; Click: AV; PAS: 4 P1’s Col.: 3 P1’s Col.: 4

P2’s Col.: 2; Click: SH; PAS: 3 P2’s Col.: 2 P2’s Col.: 2; Click: AV; PAS: ?


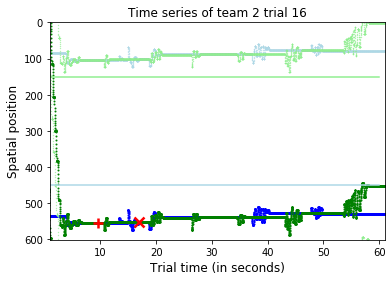

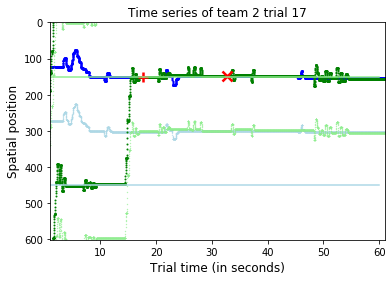

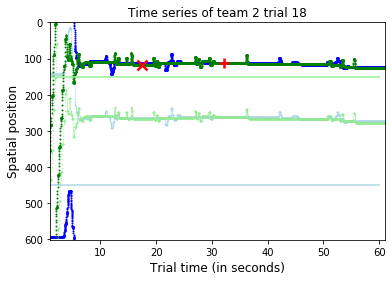


P1’s Col.: 4; Click: AV; PAS: 4 P1’s Col.: 3; Click: AV; PAS: 4 P1’s Col.: 4; Click: AV; PAS: 4

P2’s Col.: 3; Click: AV; PAS: 3 P2’s Col.: 3; Click: AV; PAS: 3 P2’s Col.: 3; Click: AV; PAS: 3


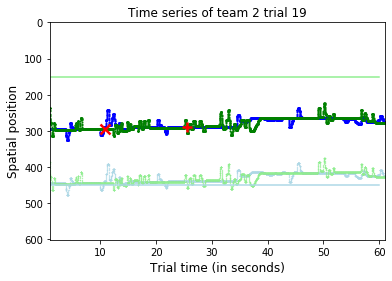

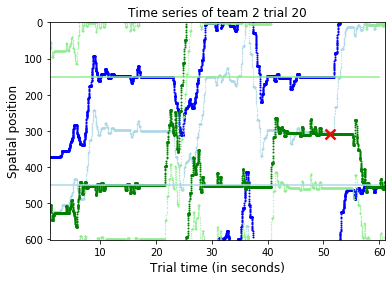


P1’s Col.: 4; Click: AV; PAS: 4 P1’s Col.: 4

P2’s Col.: 3; Click: AV; PAS: 3 P2’s Col.: 2; Click: SH; PAS: 3

***Panel 3: Team 3 (“El App”)***


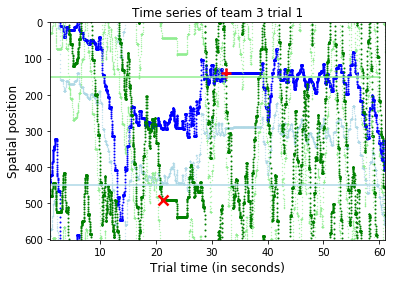

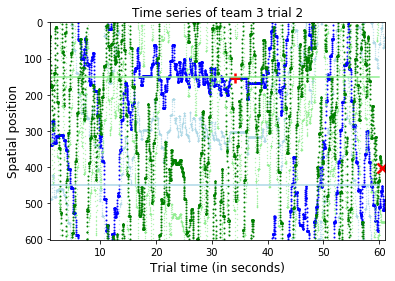

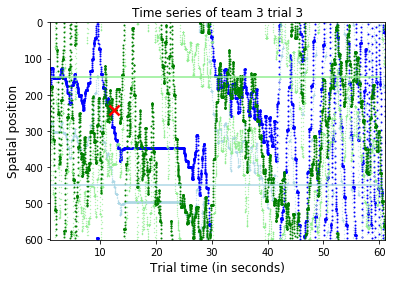


P1’s Col.: 2; Click: ST; PAS: 2 P1’s Col.: 1; Click: ST; PAS: 2 P1’s Col.: 1

P2’s Col.: 1; Click: SH; PAS: 2 P2’s Col.: 1; Click: UN; PAS: 2 P2’s Col.: 1; Click: AV; PAS: 2


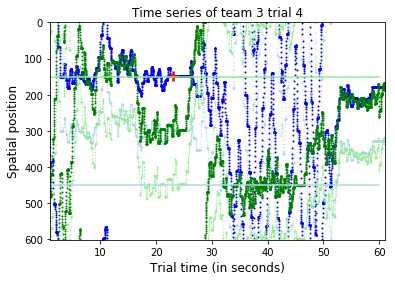

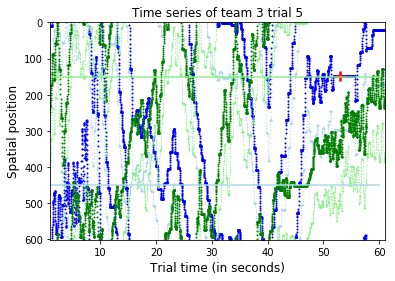

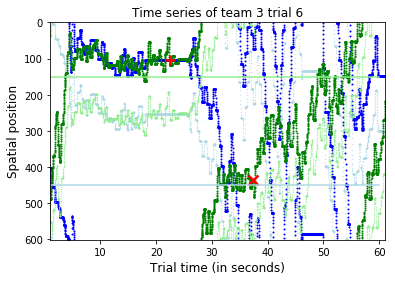


P1’s Col.: 2; Click: ST; PAS: 2 P1’s Col.: 1; Click: ST; PAS: 1 P1’s Col.: 2; Click: AV; PAS: 2

P2’s Col.: 4 P2’s Col.: 2 P2’s Col.: 3; Click: ST; PAS: 3


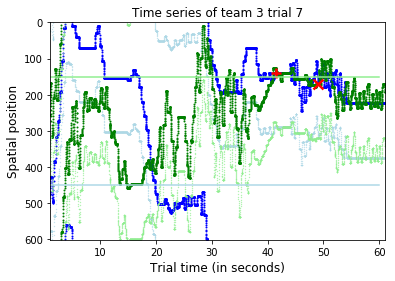

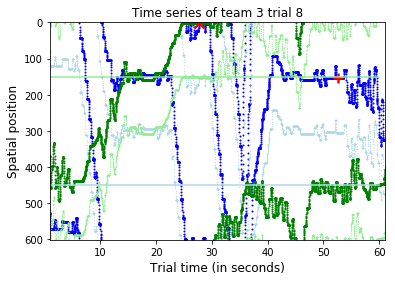

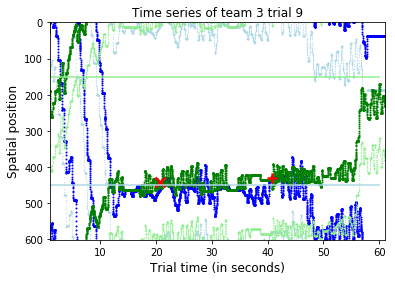


P1’s Col.: 2; Click: AV; PAS: 3 P1’s Col.: 2; Click: ST; PAS: 2 P1’s Col.: 3; Click: AV; PAS: 3

P2’s Col.: 4; Click: AV; PAS: 4 P2’s Col.: 2; Click: AV; PAS: 2 P2’s Col.: 4; Click: AV; PAS: 4


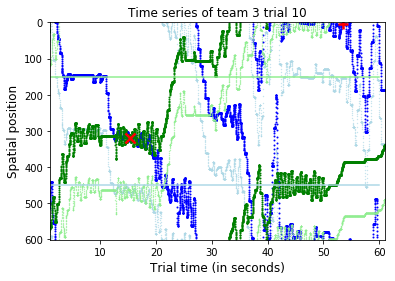

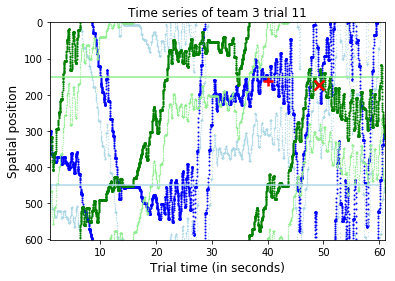

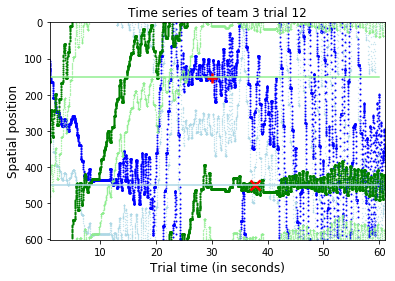


P1’s Col.: 2; Click: SH; PAS: 2 P1’s Col.: 3; Click: ST; PAS: 3 P1’s Col.: 4; Click: ST; PAS: 4

P2’s Col.: 2; Click: AV; PAS: 2 P2’s Col.: 2; Click: AV; PAS: 2 P2’s Col.: 4; Click: SH; PAS: 3


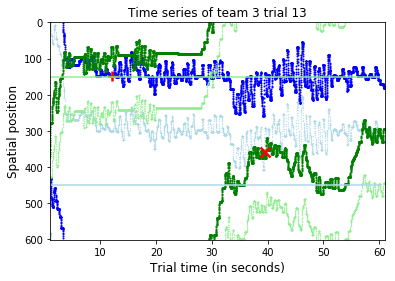

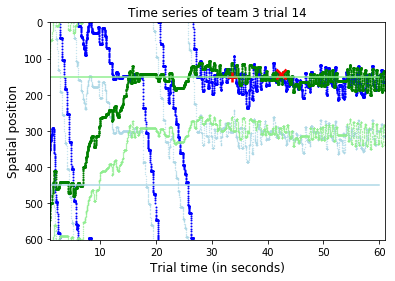

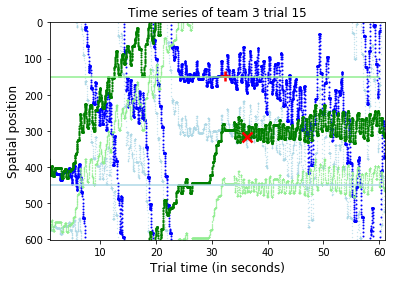


P1’s Col.: 3; Click: AV; PAS: 3 P1’s Col.: 4; Click: AV; PAS: 4 P1’s Col.: 4; Click: ST; PAS: 4

P2’s Col.: 4; Click: SH; PAS: 4 P2’s Col.: 4; Click: AV; PAS: 4 P2’s Col.: 2; Click: SH; PAS: 2


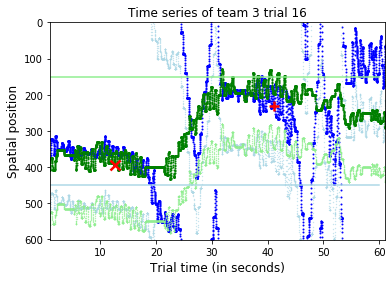

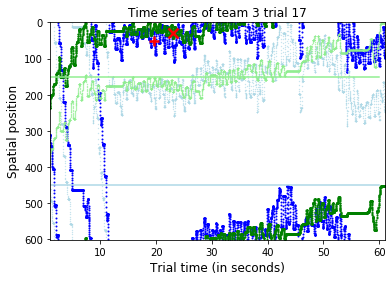

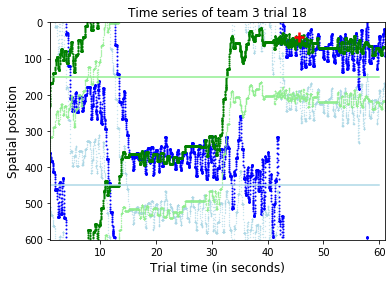


P1’s Col.: 4; Click: AV; PAS: 4 P1’s Col.: 2; Click: AV; PAS: 2 P1’s Col.: 2; Click: AV; PAS: 2

P2’s Col.: 2; Click: AV; PAS: 2 P2’s Col.: 4; Click: AV; PAS: 4 P2’s Col.: 2


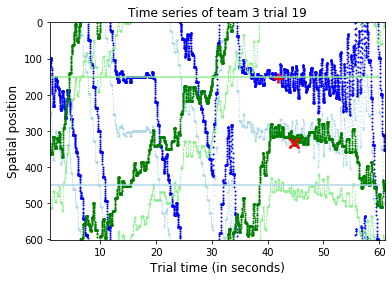

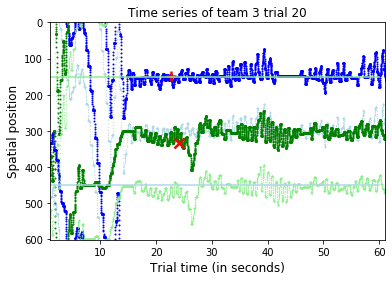


P1’s Col.: 2; Click: ST; PAS: 3 P1’s Col.: 4; Click: ST; PAS: 4

P2’s Col.: 3; Click: SH; PAS: 3 P2’s Col.: 4; Click: SH; PAS: 3

***Panel 4: Team 4 (“Emy y Ale”)***


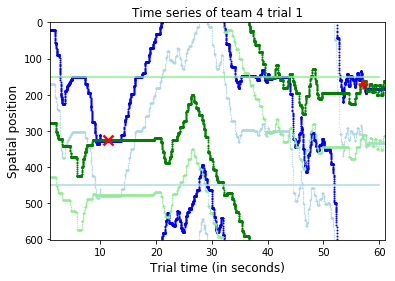

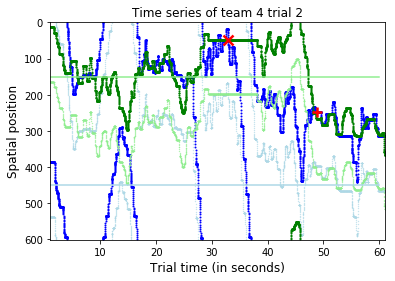

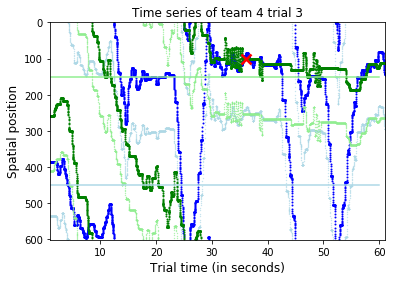


P1’s Col.: 3; Click: AV; PAS: 2 P1’s Col.: 2; Click: AV; PAS: 2 P1’s Col.: 2

P2’s Col.: 2; Click: AV; PAS: 3 P2’s Col.: 3; Click: AV; PAS: 2 P2’s Col.: 3; Click: AV; PAS: 3


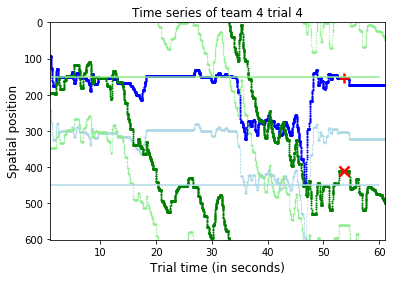

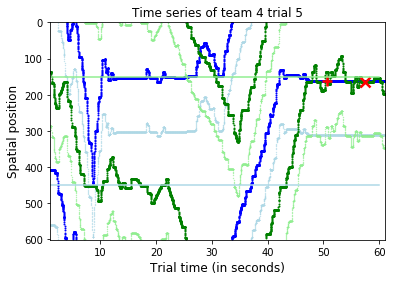

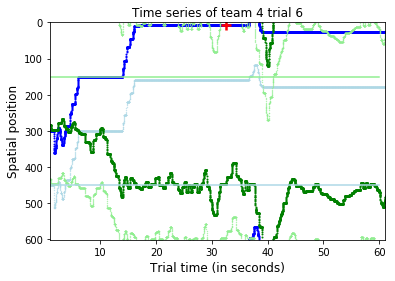


P1’s Col.: 1; Click: ST; PAS: 3 P1’s Col.: 3; Click: AV; PAS: 3 P1’s Col.: 4; Click: SH; PAS: 4

P2’s Col.: 2; Click: ST; PAS: 2 P2’s Col.: 1; Click: AV; PAS: 1 P2’s Col.: 2


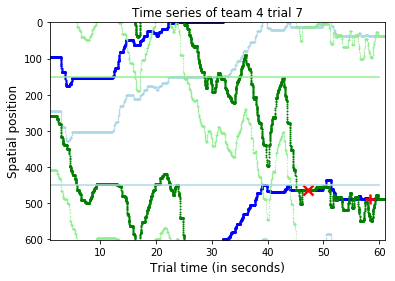

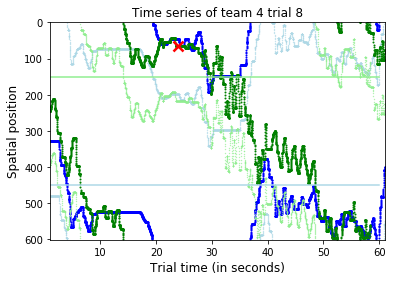

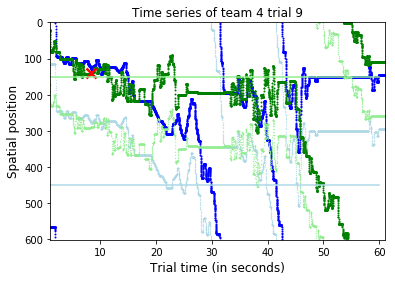


P1’s Col.: 4; Click: AV; PAS: 3 P1’s Col.: 2 P1’s Col.: 2

P2’s Col.: 4; Click: AV; PAS: 4 P2’s Col.: 4; Click: AV; PAS: 4 P2’s Col.: 4; Click: AV; PAS: 4


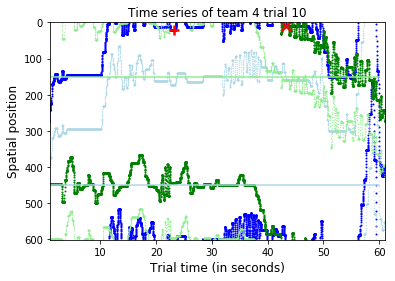

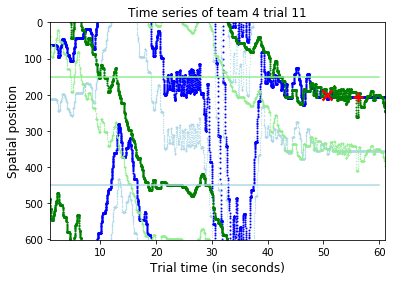

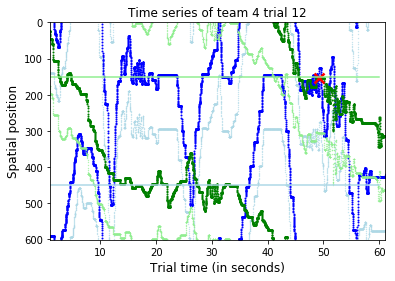


P1’s Col.: 3; Click: SH; PAS: 3 P1’s Col.: 3; Click: AV; PAS: 4 P1’s Col.: 2

P2’s Col.: 3; Click: AV; PAS: 3 P2’s Col.: 3; Click: AV; PAS: 2 P2’s Col.: 4; Click: AV; PAS: 2


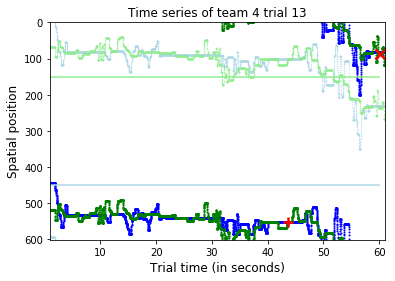

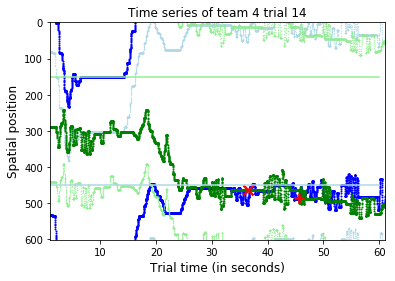

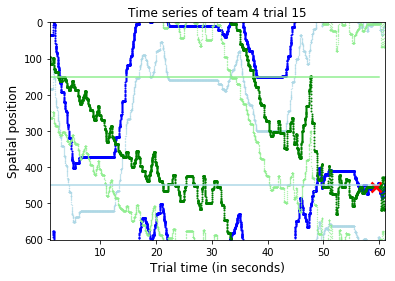


P1’s Col.: 3; Click: AV; PAS: 4 P1’s Col.: 4; Click: AV; PAS: 4 P1’s Col.: 2

P2’s Col.: 4; Click: AV; PAS: 2 P2’s Col.: 4; Click: AV; PAS: 3 P2’s Col.: 3; Click: AV; PAS: 2


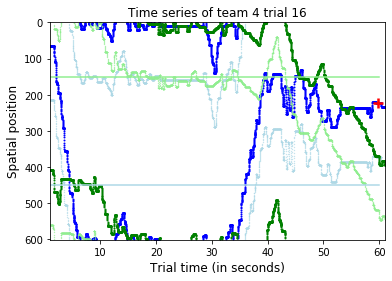

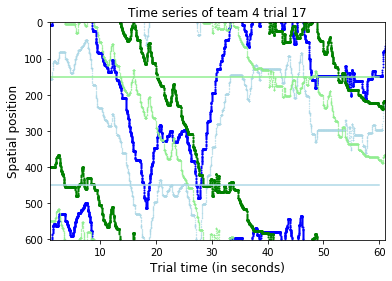

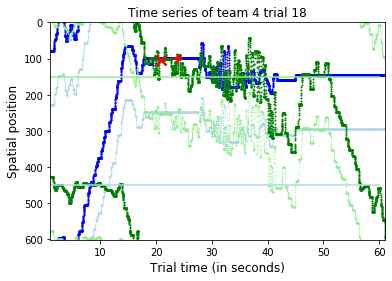


P1’s Col.: 2; Click: UN; PAS: 2 P1’s Col.: 2 P1’s Col.: 3; Click: AV; PAS: 3

P2’s Col.: 2 P2’s Col.: 2 P2’s Col.: 2; Click: AV; PAS: 2


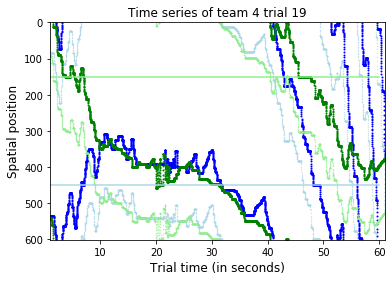

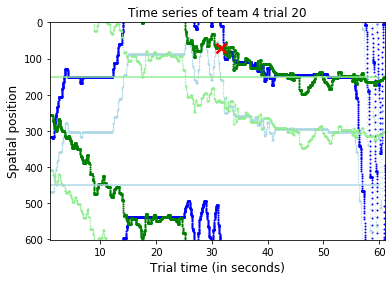


P1’s Col.: 2 P1’s Col.: 1

P2’s Col.: 1 P2’s Col.: 3; Click: SH; PAS: 2

***Panel 5: Team 5 (“Medicos”)***


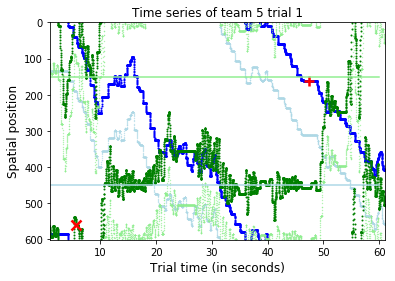

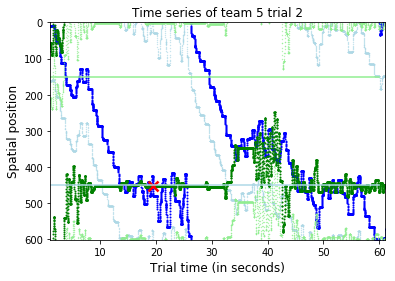

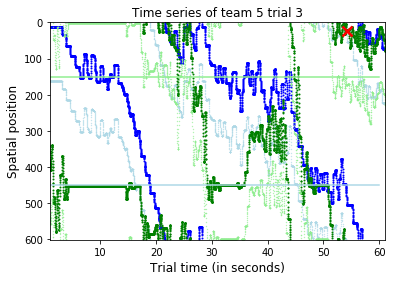


P1’s Col.: 2; Click: ST; PAS: 2 P1’s Col.: ? P1’s Col.: 2

P2’s Col.: 4; Click: AV; PAS: 4 P2’s Col.: 4; Click: AV; PAS: 4 P2’s Col.: 1; Click: SH; PAS: 2


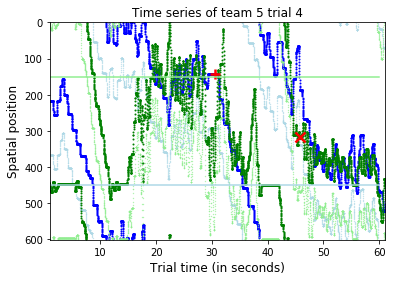

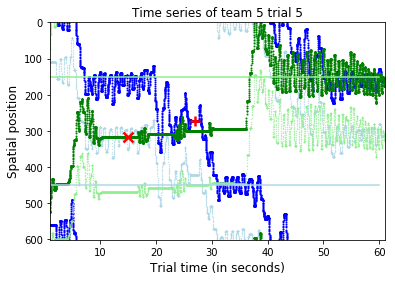

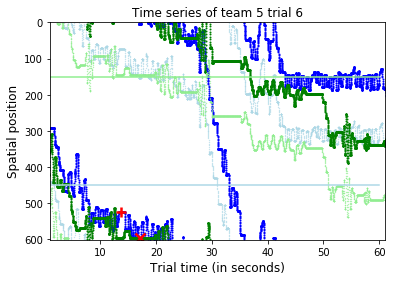


P1’s Col.: 2; Click: AV; PAS: 2 P1’s Col.: 3; Click: AV; PAS: 3 P1’s Col.: 4; Click: AV; PAS: 4

P2’s Col.: 2; Click: SH; PAS: 2 P2’s Col.: 3; Click: SH; PAS: 3 P2’s Col.: 4; Click: AV; PAS: 4


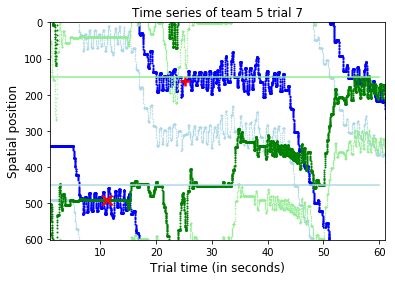

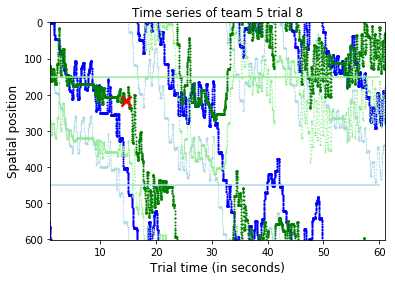

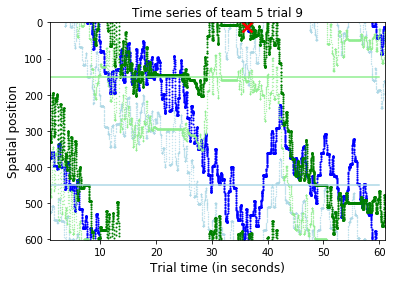


P1’s Col.: 2; Click: SH; PAS: 3 P1’s Col.: 2 P1’s Col.: 1

P2’s Col.: 4; Click: AV; PAS: 4 P2’s Col.: 4; Click: AV; PAS: 3 P2’s Col.: 4; Click: SH; PAS: 4


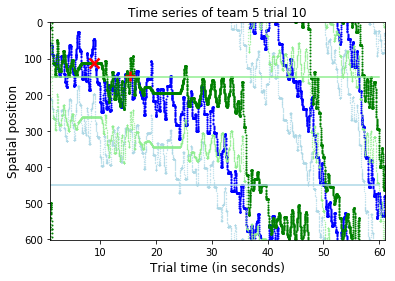

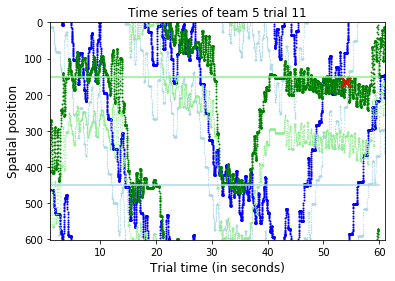

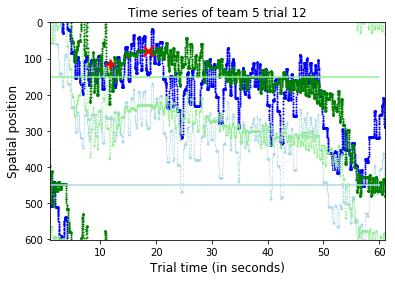


P1’s Col.: 3; Click: AV; PAS: 2 P1’s Col.: 1 P1’s Col.: 4; Click: AV; PAS: 4

P2’s Col.: 2; Click: AV; PAS: 4 P2’s Col.: 2; Click: AV; PAS: 2 P2’s Col.: 4; Click: AV; PAS: 4


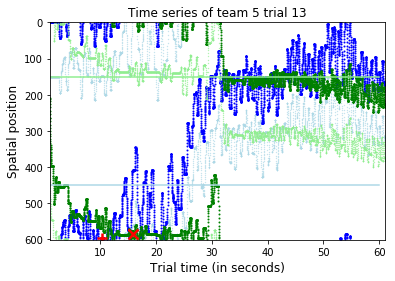

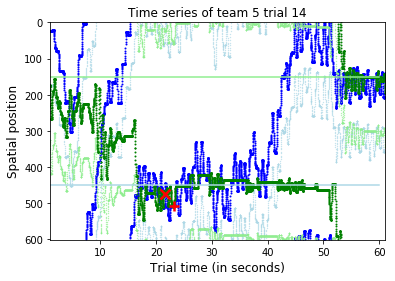

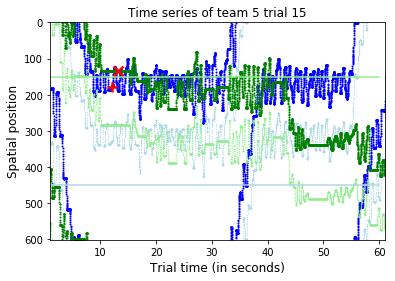


P1’s Col.: 4; Click: AV; PAS: 4 P1’s Col.: 4; Click: AV; PAS: 4 P1’s Col.: 4; Click: AV; PAS: 4

P2’s Col.: 4; Click: AV; PAS: 4 P2’s Col.: 4; Click: AV; PAS: 4 P2’s Col.: 4; Click: AV; PAS: 4


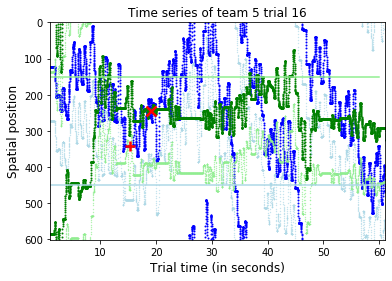

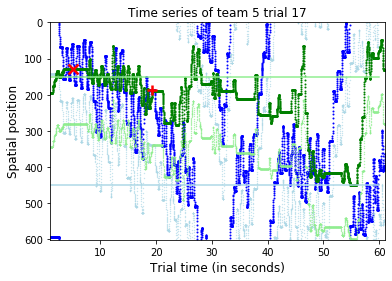

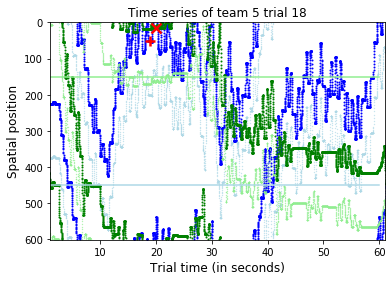


P1’s Col.: 4; Click: AV; PAS: 4 P1’s Col.: 4; Click: AV; PAS: 4 P1’s Col.: 4; Click: AV; PAS: 4

P2’s Col.: 4; Click: AV; PAS: 4 P2’s Col.: 4; Click: AV; PAS: 4 P2’s Col.: 4; Click: AV; PAS: 3


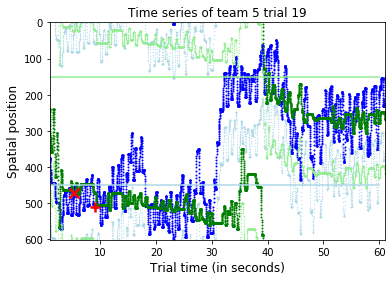

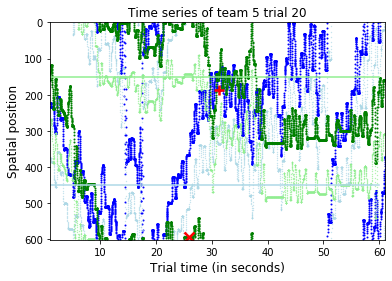


P1’s Col.: 4; Click: AV; PAS: 4 P1’s Col.: 4; Click: AV; PAS: 4

P2’s Col.: 4; Click: AV; PAS: 3 P2’s Col.: 3; Click: SH; PAS: 4

***Panel 6: Team 6 (“Redes”)***


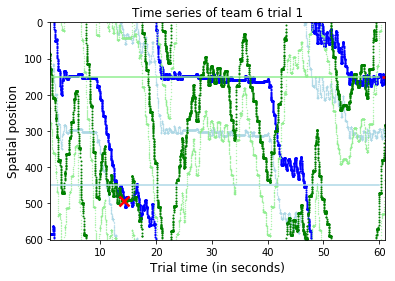

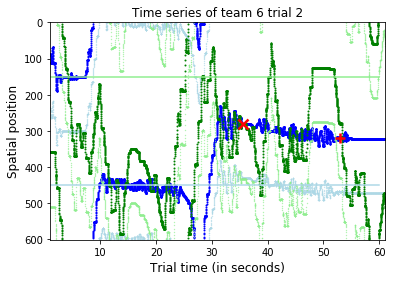

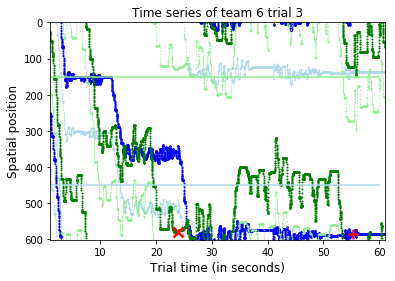


P1’s Col.: 2; Click: ST; PAS: 3 P1’s Col.: 3; Click: AV; PAS: 4 P1’s Col.: 4; Click: SH; PAS: 4

P2’s Col.: 1; Click: AV; PAS: 1 P2’s Col.: 2; Click: AV; PAS: 2 P2’s Col.: 2; Click: SH; PAS: 2


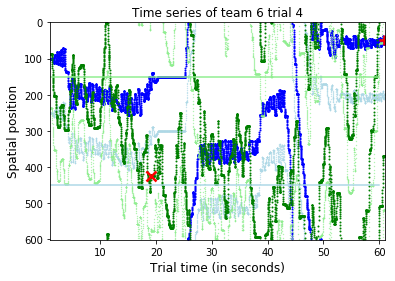

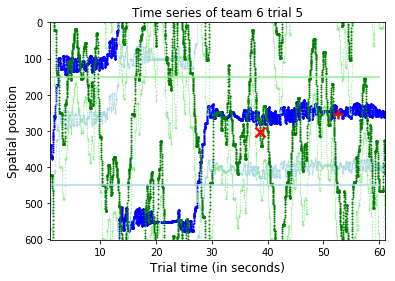


P1’s Col.: 4; Click: SH; PAS: 4 P1’s Col.: 4; Click: AV; PAS: 4 P1’s Col.: 2

P2’s Col.: 3; Click: SH; PAS: 3 P2’s Col.: 2; Click: AV; PAS: 2 P2’s Col.: 1; Click: ST; PAS: 2

P1’s Col.: 4; Click: AV; PAS: 4 P1’s Col.: 3; Click: AV; PAS: 4 P1’s Col.: 2; Click: AV; PAS: 2

P2’s Col.: 3; Click: AV; PAS: 3 P2’s Col.: 1; Click: AV; PAS: 2 P2’s Col.: 2; Click: AV; PAS: 2

P1’s Col.: 2 P1’s Col.: 2; Click: AV; PAS: 4 P1’s Col.: 3; Click: AV; PAS: 3

P2’s Col.: 2; Click: SH; PAS: 3 P2’s Col.: 2; Click: AV; PAS: 3 P2’s Col.: 1; Click: AV; PAS: 1

P1’s Col.: ?; Click: SH; PAS: ? P1’s Col.: 2; Click: AV; PAS: 3 P1’s Col.: 4; Click: AV; PAS: 4

P2’s Col.: ?; Click: AV; PAS: ? P2’s Col.: 3; Click: SH; PAS: 3 P2’s Col.: 3

P1’s Col.: 1; Click: AV; PAS: 4 P1’s Col.: 4 P1’s Col.: 2; Click: AV; PAS: 4

P2’s Col.: 2; Click: AV; PAS: 3 P2’s Col.: 4 P2’s Col.: 3; Click: AV; PAS: 3

P1’s Col.: 4; Click: AV; PAS: ?

P2’s Col.: 4; Click: AV; PAS: 3

***Panel 7: Team 7 (“Reta jungla”)***

P1’s Col.: 2; Click: AV; PAS: 2 P1’s Col.: 2; Click: ST; PAS: 2 P1’s Col.: 2; Click: SH; PAS: 2

P2’s Col.: 2; Click: AV; PAS: 2 P2’s Col.: 2; Click: AV; PAS: 2 P2’s Col.: 2; Click: AV; PAS: 2

P1’s Col.: 2; Click: AV; PAS: 2 P1’s Col.: 3; Click: SH; PAS: 3 P1’s Col.: 3; Click: ST; PAS: 3

P2’s Col.: 2; Click: AV; PAS: 2 P2’s Col.: 2; Click: AV; PAS: 2 P2’s Col.: 1; Click: AV; PAS: 1

P1’s Col.: 3; Click: AV; PAS: 3 P1’s Col.: 3; Click: SH; PAS: 2 P1’s Col.: 2; Click: AV; PAS: 2

P2’s Col.: 2; Click: AV; PAS: 3 P2’s Col.: 2 P2’s Col.: 3

P1’s Col.: 3; Click: AV; PAS: 3 P1’s Col.: 3; Click: AV; PAS: 3 P1’s Col.: 3; Click: AV; PAS: 2

P2’s Col.: 3; Click: AV; PAS: 3 P2’s Col.: 3; Click: SH; PAS: 3 P2’s Col.: 3

P1’s Col.: 3; Click: AV; PAS: 3 P1’s Col.: 3; Click: AV; PAS: 3 P1’s Col.: 4; Click: ST; PAS: 4

P2’s Col.: 3 P2’s Col.: 3 P2’s Col.: 3; Click: AV; PAS: 3

P1’s Col.: 2; Click: AV; PAS: 2 P1’s Col.: 2; Click: AV; PAS: 1 P1’s Col.: 2; Click: AV; PAS: 2

P2’s Col.: 3; Click: AV; PAS: 3 P2’s Col.: 3 P2’s Col.: 3; Click: AV; PAS: 3

P1’s Col.: 2; Click: AV; PAS: P1’s Col.: 3; Click: AV; PAS: 2

P2’s Col.: 3 P2’s Col.: 3; Click: AV; PAS: 3

***Panel 8: Team 8 (“Siniestros”)***

P1’s Col.: 4; Click: AV; PAS: 4 P1’s Col.: 3; Click: AV; PAS: 3 P1’s Col.: 3; Click: AV; PAS: 3

P2’s Col.: 3; Click: AV; PAS: 2 P2’s Col.: 3; Click: SH; PAS: 3 P2’s Col.: 3

P1’s Col.: 4; Click: AV; PAS: 4 P1’s Col.: 2 P1’s Col.: 4; Click: AV; PAS: 4

P2’s Col.: 4; Click: AV; PAS: 4 P2’s Col.: 2; Click: SH; PAS: 2 P2’s Col.: 2; Click: AV; PAS: 2

P1’s Col.: 4; Click: AV; PAS: 4 P1’s Col.: 4; Click: AV; PAS: 4 P1’s Col.: 2; Click: AV; PAS: 2

P2’s Col.: 4; Click: AV; PAS: 4 P2’s Col.: 3 P2’s Col.: 3

P1’s Col.: 4; Click: AV; PAS: 4 P1’s Col.: 3 P1’s Col.: 2; Click: SH; PAS: 2

P2’s Col.: 4; Click: AV; PAS: 4 P2’s Col.: 2; Click: AV; PAS: 2 P2’s Col.: 3; Click: ST; PAS: 3

P1’s Col.: 4; Click: AV; PAS: 4 P1’s Col.: 3; Click: AV; PAS: 3 P1’s Col.: 3; Click: AV; PAS: 4

P2’s Col.: 3; Click: AV; PAS: 3 P2’s Col.: 4; Click: AV; PAS: 4 P2’s Col.: 3; Click: AV; PAS: 3

P1’s Col.: 4; Click: AV; PAS: 4 P1’s Col.: 2 P1’s Col.: 4; Click: AV; PAS: 4

P2’s Col.: 4; Click: AV; PAS: 4 P2’s Col.: 3 P2’s Col.: 2; Click: AV; PAS: 2

P1’s Col.: 4; Click: AV; PAS: 3 P1’s Col.: 2; Click: SH; PAS: 2

P2’s Col.: 3; Click: AV; PAS: 3 P2’s Col.: 3; Click: ST; PAS: 3

***Panel 9: Team 9 (“Twenty Five”)***

P1’s Col.: 2; Click: AV; PAS: 2 P1’s Col.: 2; Click: AV; PAS: 1 P1’s Col.: 2; Click: ST; PAS: 2

P2’s Col.: 2; Click: AV; PAS: 2 P2’s Col.: 2; Click: AV; PAS: 2 P2’s Col.: 3; Click: AV; PAS: 3

P1’s Col.: 2; Click: AV; PAS: 2 P1’s Col.: 2; Click: SH; PAS: 3 P1’s Col.: 2; Click: UN; PAS: 1

P2’s Col.: 1 P2’s Col.: 1; Click: AV; PAS: 2 P2’s Col.: 2; Click: AV; PAS: 3

P1’s Col.: 2; Click: SH; PAS: 2 P1’s Col.: 2; Click: AV; PAS: 2 P1’s Col.: 2; Click: AV; PAS: 2

P2’s Col.: 3 P2’s Col.: 1; Click: AV; PAS: 2 P2’s Col.: 1; Click: AV; PAS: 2

P1’s Col.: 3; Click: AV; PAS: 3 P1’s Col.: 2 P1’s Col.: 2

P2’s Col.: 2; Click: AV; PAS: 3 P2’s Col.: 3; Click: AV; PAS: 4 P2’s Col.: 1

P1’s Col.: 1; Click: UN; PAS: 1 P1’s Col.: 1; Click: AV; PAS: 1 P1’s Col.: 3; Click: AV; PAS: 3

P2’s Col.: 3; Click: AV; PAS: 3 P2’s Col.: 3; Click: AV; PAS: 4 P2’s Col.: 1; Click: AV; PAS: 3

P1’s Col.: 2; Click: AV; PAS: 2 P1’s Col.: 1; Click: AV; PAS: 1 P1’s Col.: 1; Click: AV; PAS: 1

P2’s Col.: 2; Click: AV; PAS: 3 P2’s Col.: 3; Click: AV; PAS: 3 P2’s Col.: 2; Click: AV; PAS: 2

P1’s Col.: 1; Click: AV; PAS: 1 P1’s Col.: 2

P2’s Col.: 3; Click: AV; PAS: 4 P2’s Col.: 2; Click: UN; PAS: 4

***Panel 10: Team 10 (“Vibrantes”)***

P1’s Col.: 2; Click: ST; PAS: 3 P1’s Col.: 2 P1’s Col.: 1; Click: AV; PAS: 2

P2’s Col.: 3; Click: ST; PAS: 2 P2’s Col.: 2 P2’s Col.: 3; Click: ST; PAS: 3

P1’s Col.: 1; Click: AV; PAS: 1 P1’s Col.: 4; Click: AV; PAS: 4 P1’s Col.: 3

P2’s Col.: 2 P2’s Col.: 2 P2’s Col.: 1; Click: ST; PAS: 1

P1’s Col.: 3; Click: AV; PAS: 3 P1’s Col.: 2; Click: UN; PAS: 3 P1’s Col.: 2; Click: UN; PAS: 2

P2’s Col.: 2; Click: ST; PAS: 2 P2’s Col.: 4 P2’s Col.: 3

P1’s Col.: 2; Click: UN; PAS: 3 P1’s Col.: 3; Click: AV; PAS: 3 P1’s Col.: 4; Click: ST; PAS: 4

P2’s Col.: 2 P2’s Col.: 1 P2’s Col.: 1; Click: ST; PAS: 1

P1’s Col.: 4 P1’s Col.: 4 P1’s Col.: 4; Click: UN; PAS: 4

P2’s Col.: 2; Click: AV; PAS: 2 P2’s Col.: 4; Click: AV; PAS: 4 P2’s Col.: 4

P1’s Col.: 2; Click: UN; PAS: 3 P1’s Col.: 4 P1’s Col.: 4

P2’s Col.: 4; Click: AV; PAS: 4 P2’s Col.: 4 P2’s Col.: 2; Click: AV; PAS: 2

P1’s Col.: 2 P1’s Col.: 3; Click: UN; PAS: 3

P2’s Col.: 4 P2’s Col.: 1; Click: AV; PAS: 1

**Supplementary Figure 1.** Plots of trajectories and clicks during each trial. Participant 1 (P1) can encounter all green objects, i.e. the avatar and the shadow of participant 2 (P2) and the static object at position 150, while P2 can encounter all blue objects, i.e. the avatar and the shadow of P1 and the static object at position 450. P1’s clicks are indicated on top of P1’s trajectory (blue line) with a red ‘+’, while P2’s clicks are indicated on P2’s trajectory (green line) with a red ‘x’.

**References**

Froese, T., Iizuka, H., & Ikegami, T. (2014). Embodied social interaction constitutes social cognition in pairs of humans: A minimalist virtual reality experiment. *Scientific Reports*, 4(3672). doi:10.1038/srep03672

Hermans, K. S. F. M., Kasanova, Z., Zapata-Fonseca, L., Lafit, G., Fossion, R., Froese, T., & Myin-Germeys, I. (in press). Investigating real-time social interaction in pairs of adolescents with the perceptual crossing experiment. *Behavior Research Methods*.

Kojima, H., Froese, T., Oka, M., Iizuka, H., & Ikegami, T. (2017). A sensorimotor signature of the transition to conscious social perception: Co-regulation of active and passive touch. *Frontiers in Psychology*, 8, 1778. doi:10.3389/fpsyg.2017.01778
