# Supplementary material for: The Feeling Is Mutual: Clarity of Haptics-Mediated Social Perception Is Not Associated With the Recognition of the Other, Only With Recognition of Each Other
Source: Front Hum Neurosci. 2020 Sep 4;14:560567. doi: 10.3389/fnhum.2020.560567 (PMC7500513; doi:10.3389/fnhum.2020.560567)
Supplement: Supplementary file 2 [file Data_Sheet_1.PDF]

Supplementary Statistical Information

1 DATA

The data for the statistical analysis are the responses of both players in each of 10 dyads for each of 20 trials in the Perceptual Crossing Task. In this document, we will use the index i ($i = 1, \ldots, 10$) to refer to the dyads and the index j ($j = 1, \ldots, 20$) to refer to the trials within a dyad; the index k ($k = 1, 2$) refers to the players within a dyad.

We consider two types of responses by each player in each trial:

- Recognizing the other player by giving a correct click: We define X_{ijk} as a dichotomous variable which assumes a value of 1 if player k of dyad i made a correct click (i.e., a click in response of crossing the avatar of the other player) during trial j; and 0 otherwise (i.e., clicking in response of another event or not producing a click).
- The rating given on the Perceptual Awareness Scale (PAS): Y_{ijk} is an ordinal variable with four categories that indicate the level of a player's awareness of the presence of the other player during the interaction (viz., just before giving a click). Here, we code Y_{ijk}, the PAS-response of player k of dyad i for trial j, in the following way:

$$Y_{ijk} = \begin{cases} 0 & \text{if the player indicates ``having had no experience of the other'',} \\ 1 & \text{if the player indicates ``having had an ambiguous experience of the other'',} \\ 2 & \text{if the player indicates ``having had an almost clear experience of the other'',} \\ 3 & \text{if the player indicates ``having had a clear experience of the other''.} \end{cases}$$

Note that Y_{ijk} is missing whenever the individual did not produce a click during the trial.

Furthermore, at the level of the dyad, the following variables are introduced:

- Short inter-click delay: The binary variable D_{ij} assumes a value of 1, if both players of dyad i clicked during trial j within a time frame of 3 seconds or less, and 0 otherwise (which includes the case that either of both players did not click). This definition applies irrespective of whether the players produced a correct click or not.
- Jointly recognizing the other player:

$$XX_{ij} = X_{ij1} \times X_{ij2}.$$

The data are available in tabular format in the appendix of this document. One may note that the experiment for the first dyad lasted only 15 trials and that for the sixth dyad data for the 20th trial were not registered due to a technical problem.

2 MODEL

We simultaneously modeled the relations among the variables defined in the previous section as depicted in Figure S1. The model implies three endogenous variables (i.e., variables which are affected by other

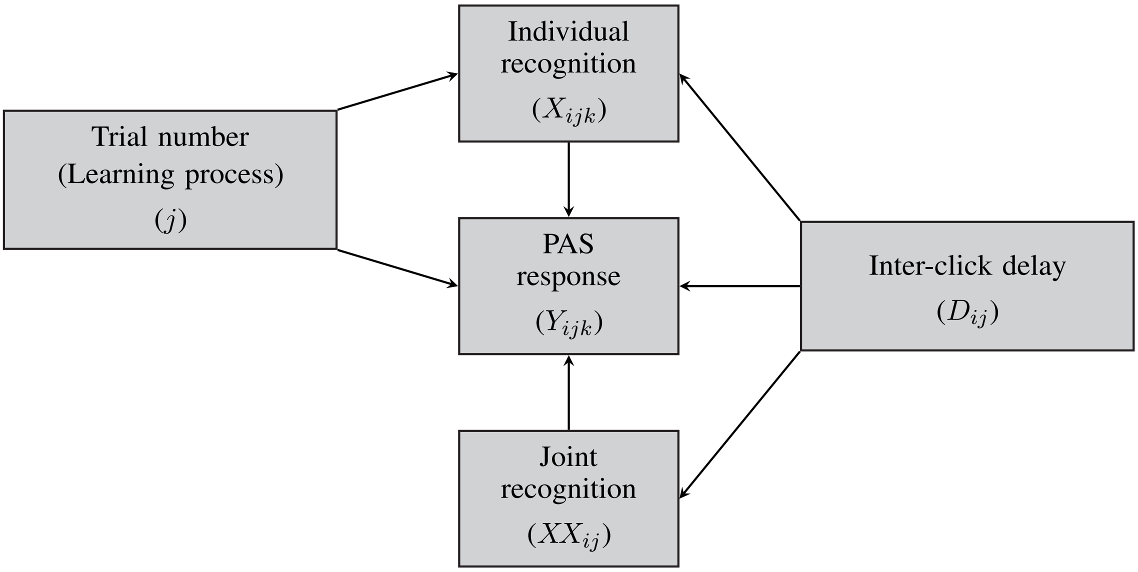

Figure S1. Relations among the observed variables specified by the statistical model.

variables in the model). In the following subsections, we provide the details of the sub-models of these variables: The sub-model for joint recognition (XX_{ij}; Section 2.1), individual recognition (X_{ijk}; Section 2.2), and the PAS-response (Y_{ijk}; Section 2.3).

2.1 Sub-model for joint success

A standard probit regression model is assumed for the binary variable XX_{ij}, with D_{ij} as explicative (or independent) variable. That is, for all i and j, we have:

$$\Pr(XX_{ij} = 1) \;=\; \Phi\left(\beta_0^{(XX)} \;+\; \beta_D^{(XX)} D_{ij}\right), \tag{S1}$$

where $\Phi(x)$ denotes the standard normal cumulative distribution function evaluated at x; $\beta_0^{(XX)}$ and $\beta_D^{(XX)}$ are regression parameters that will be estimated from the data.

2.2 Sub-model for individual success

Whereas the variable XX_{ij} is located at the dyad/trial level, X_{ijk} is at the individual/trial level. In order to take into account the association between X_{ij1} and X_{ij2}, we specified a *bivariate* probit regression model for these variables. In particular, we modeled their joint probability by assuming underlying continuous variables $X^\star_{ij1}$ and $X^\star_{ij2}$, which have a bivariate normal distribution (with, for identifiability reasons, means and variances set to 0 and 1, respectively):

$$(X^\star_{ij1}, X^\star_{ij2}) \overset{\text{iid}}{\sim} \text{Normal}\left[\begin{pmatrix}0\\0\end{pmatrix}, \begin{pmatrix}1 & \rho_{XX}\\ \rho_{XX} & 1\end{pmatrix}\right]. \tag{S2}$$

The parameter ρ_{XX}, which can be interpreted as the correlation between both players' abilities of recognizing the other, will be estimated from the data. In a probit model, the relation between the observed X_{ijk} and the latent $X^\star_{ijk}$ is through a threshold $\tau^{(X)}_{ijk}$, which satisfies for all i, j, and k:

$$X_{ijk} \;=\; 1 \quad\Longleftrightarrow\quad X^\star_{ijk} < \tau^{(X)}_{ijk}.$$

As a result, the joint probability distribution for (X_{ij1}, X_{ij2}) can be calculated under the bivariate normal distribution for $(X^\star_{ij1}, X^\star_{ij2})$. For example,

$$\begin{aligned}\Pr(X_{ij1} = 1 \text{ and } X_{ij2} = 0) &= \Pr\left(X^\star_{ij1} < \tau^{(X)}_{ij1} \text{ and } X^\star_{ij2} \geqslant \tau^{(X)}_{ij2}\right) \\ &= \int_{-\infty}^{\tau^{(X)}_{ij1}} \int_{\tau^{(X)}_{ij2}}^{\infty} \frac{1}{2\pi\sqrt{1-\rho^2_{XX}}} \exp\left[-\frac{x_1^2 + x_2^2 - 2\rho_{XX}x_1x_2}{2(1-\rho^2_{XX})}\right] \mathrm{d}x_2 \mathrm{d}x_1. \end{aligned} \quad \text{(S3)}$$

The thresholds $\tau^{(X)}_{ijk}$ thus determine the probability of individual success; this threshold then is further assumed to be affected by a learning process and the inter-click delay (see Figure S1), as specified in the following equation:

$$\tau^{(X)}_{ijk} = \beta^{(X)}_{0ik} + \beta^{(X)}_{1ik} Z^{(X)}_{ijk} + \beta^{(X)}_{D} D_{ij}, \quad \text{(S4)}$$

where

- $Z^{(X)}_{ijk}$ is defined as:

$$\begin{cases} Z^{(X)}_{ijk} = 0 & \text{if } j \geqslant \zeta^{(X)}_{ik}, \\ Z^{(X)}_{ijk} = j - \zeta^{(X)}_{ik} & \text{if } j < \zeta^{(X)}_{ik}. \end{cases}$$

- The $\beta^{(X)}_{0ik}$, $\beta^{(X)}_{1ik}$, and $\zeta^{(X)}_{ik}$ are parameters that model (piece-wise linearly) the evolution of the threshold $\tau^{(X)}_{ijk}$ (i.e., the learning process) for individual k of dyad i across different trials j. The evolution is assumed to consist of two stages: During the first stage, from trial 1 to trial $\zeta^{(X)}_{ik}$, which can be interpreted as a learning stage, $\tau^{(X)}_{ijk}$ increases linearly, with a slope equal to $\beta^{(X)}_{1ik}$; then, during the consolidation stage, which starts at the breakpoint $\zeta^{(X)}_{ik}$, $\tau^{(X)}_{ijk}$ remains constant at the value $\beta^{(X)}_{0ik}$.
- $\beta^{(X)}_{D}$ denotes the additional increase of the threshold $\tau^{(X)}_{ijk}$ when the inter-click delay between both players is below 3 seconds.

As implied by the above description of the model, the parameters $\beta^{(X)}_{0ik}$, $\beta^{(X)}_{1ik}$, and $\zeta^{(X)}_{ik}$ are random effects, as they are allowed to vary across individuals. The following distributions are assumed for these random effects:

$$\beta^{(X)}_{0ik} \overset{\text{iid}}{\sim} \text{Normal}\left(\mu^{(X)}_0, \sigma^{2(X)}_0\right), \quad \text{(S5a)}$$

$$\beta^{(X)}_{1ik} \overset{\text{iid}}{\sim} \text{Normal}\left(\mu^{(X)}_1, \sigma^{2(X)}_1\right), \quad \text{(S5b)}$$

$$\zeta^{(X)}_{ik} \overset{\text{iid}}{\sim} \text{Beta}\left(\alpha^{(X)}, \beta^{(X)}, 1, 20\right). \quad \text{(S5c)}$$

This means that $\mu^{(X)}_0$ and $\sigma^{2(X)}_0$ denote the mean and variance across individuals of the level reached after the learning stage. Likewise, $\mu^{(X)}_1$ and $\sigma^{2(X)}_1$ are the mean and variance of the increase (per trial, on the probit scale) during the learning stage. Note that the break points $\zeta^{(X)}_{ik}$ follow a four-parameter beta distribution, with support in the range $[1, 20]$. The parameters of the latter distributions, as well as the random effects themselves, are estimated from the data.

2.3 Sub-model for the PAS-response

A similar approach as the one outlined in the previous subsection was adopted for the variable Y_{ijk}. However, contrary to X_{ijk}, which only takes values 0 and 1, Y_{ijk} is a polytomous variable with ordered categories. Hence, we specified a bivariate *ordinal* probit regression model for the PAS-responses given by the two members of dyad i at trial j, that is, for the pair (Y_{ij1}, Y_{ij2}). This implies again that we assume latent continuous variables $Y^\star_{ij1}$ and $Y^\star_{ij2}$, with a bivariate normal distribution:

$$(Y^\star_{ij1}, Y^\star_{ij2}) \overset{\text{iid}}{\sim} \text{Normal}\left[\begin{pmatrix}0\\0\end{pmatrix}, \begin{pmatrix}1 & \rho_{YY}\\ \rho_{YY} & 1\end{pmatrix}\right], \tag{S6}$$

with the parameter ρ_{YY}, the correlation between the (latent continuous) PAS-responses of both members of a dyad, to be estimated from the data. The main difference with the sub-model for X_{ijk} is the use of three, rather than one, thresholds to relate the $Y^\star_{ijk}$ and Y_{ijk}:

$$Y_{ijk} = \begin{cases} 0 & \iff \tau_{1ijk} \leqslant Y^\star_{ijk}, \\ 1 & \iff \tau_{2ijk} \leqslant Y^\star_{ijk} < \tau^{(Y)}_{1ijk}, \\ 2 & \iff \tau_{3ijk} \leqslant Y^\star_{ijk} < \tau^{(Y)}_{2ijk}, \\ 3 & \iff Y^\star_{ijk} < \tau^{(Y)}_{3ijk}. \end{cases}$$

That is, we have three ordered thresholds ($\tau^{(Y)}_{1ijk} > \tau^{(Y)}_{2ijk} > \tau^{(Y)}_{3ijk}$) that divide the latent dimension in four regions, each corresponding to one of the four observed ordered categories. Then, the joint probability for (Y_{ij1}, Y_{ij2}) can be calculated under the bivariate normal distribution in (S6), along the same principles as exemplified in Equation (S3).[1]

We further assume constant differences among the thresholds, across all dyads, individuals, and trials. In particular, for all i, j, and k, we specify:

$$\tau^{(Y)}_{1ijk} - \tau^{(Y)}_{2ijk} = \delta_{12} \qquad \left(\iff \tau^{(Y)}_{2ijk} = \tau^{(Y)}_{1ijk} - \delta_{12}\right) \tag{S7a}$$

$$\tau^{(Y)}_{2ijk} - \tau^{(Y)}_{3ijk} = \delta_{23} \qquad \left(\iff \tau^{(Y)}_{3ijk} = \tau^{(Y)}_{2ijk} - \delta_{23} = \tau^{(Y)}_{1ijk} - \delta_{12} - \delta_{23}\right). \tag{S7b}$$

In these equations, δ_{12} and δ_{23} are parameters that will be estimated from the data. The assumption of constant differences means that the psychological distances between the ordered PAS-ratings are assumed to be constant across persons and trials. One may note that this is a common assumption in this kind of models.

As the thresholds $\tau^{(Y)}_{2ijk}$ and $\tau^{(Y)}_{3ijk}$ are completely determined by $\tau^{(Y)}_{1ijk}$ (and the fixed parameters δ_{12} and δ_{23}), we now focus on the model for the first threshold. The approach taken is almost identical as for $\tau^{(X)}_{ijk}$ in the previous subsection. Following Figure S1, we specify the following regression model for $\tau^{(Y)}_{1ijk}$:

$$\tau^{(Y)}_{1ijk} = \beta^{(Y)}_{0ik} + \beta^{(Y)}_{1ik}\, Z^{(Y)}_{ijk} + \beta^{(Y)}_{X}\, X_{ijk} + \beta^{(Y)}_{XX}\, XX_{ij} + \beta^{(Y)}_{D}\, D_{ij}, \tag{S8}$$

where

[1] As explained in Section 1, Y_{ijk} can be missing; in that case, the joint probability for (Y_{ij1}, Y_{ij2}) reduces to the marginal probability considering only the response of the other player (i.e., if Y_{ij1} is missing, we calculate the marginal probability for Y_{ij2}, which is based on the standard normal distribution for $Y^\star_{ij2}$. If both Y_{ij1} and Y_{ij2} are missing, the probability is fixed to 1. This approach is in line with assuming that the responses are MAR (missing at random; see Rubin, 1976).

- $Z^{(Y)}_{ijk}$ is defined as:

$$\begin{cases} Z^{(Y)}_{ijk} = 0 & \text{if } j \geqslant \zeta^{(Y)}_{ik}, \\ Z^{(Y)}_{ijk} = j - \zeta^{(Y)}_{ik} & \text{if } j < \zeta^{(Y)}_{ik}. \end{cases}$$

- The $\beta^{(Y)}_{0ik}$, $\beta^{(Y)}_{1ik}$, and $\zeta^{(Y)}_{ik}$ model a piece-wise linear learning process for the (latent) PAS-response, in a complete analogous way as the learning process for individual success (assumed for threshold $\tau^{(X)}_{ijk}$ in Equation (S4)). Note that we are assuming separate learning processes for individual success in recognizing the other player (X_{ijk} and $\tau^{(X)}_{ijk}$) and the perceptual awareness of the other player (Y_{ijk} and $\tau^{(Y)}_{1ijk}$).
- $\beta^{(Y)}_{X}$, $\beta^{(Y)}_{XX}$, and $\beta^{(Y)}_{D}$ denote the effect of individual success, joint success, and a short interclick delay, respectively, on the threshold $\tau^{(Y)}_{1ijk}$ (and, hence, on the probability of responding in each of the four reponse categories of the PAS-question).

Whereas the latter parameters ($\beta^{(Y)}_{X}$, $\beta^{(Y)}_{XX}$, and $\beta^{(Y)}_{D}$) are considered fixed, the $\beta^{(Y)}_{0ik}$, $\beta^{(Y)}_{1ik}$, and $\zeta^{(Y)}_{ik}$ are allowed to vary across individuals. That is, they are random-effects parameters, for which we assume the following distributions:

$$\beta^{(Y)}_{0ik} \overset{\text{iid}}{\sim} \text{Normal}\left(\mu^{(Y)}_0, \sigma^{2(Y)}_0\right), \tag{S9a}$$

$$\beta^{(Y)}_{1ik} \overset{\text{iid}}{\sim} \text{Normal}\left(\mu^{(Y)}_1, \sigma^{2(Y)}_1\right), \tag{S9b}$$

$$\zeta^{(Y)}_{ik} \overset{\text{iid}}{\sim} \text{Beta}\left(\alpha^{(Y)}, \beta^{(Y)}, 1, 20\right), \tag{S9c}$$

with interpretations similar to the interpretations for the corresponding parameters as in the equations (S5).

3 ESTIMATION

To estimate the parameters of the model specified in the previous section, we adopted a Bayesian framework. In a Bayesian analysis, one is interested in the posterior distribution of the parameters (Gelman, Carlin, Stern, Dunson, Vehtari, and Rubin, 2014). The posterior distribution summarizes the plausible values for the parameters given the observed data and, by Bayes theorem, is proportional to the product of the likelihood of the data and the prior distribution of the parameters:

$$f(\boldsymbol{\theta} \,|\, \mathbf{y}) \;\propto\; f(\mathbf{y} \,|\, \boldsymbol{\theta})\, f(\boldsymbol{\theta}),$$

where $\boldsymbol{\theta}$ denotes the vector of all parameters in the model and $\mathbf{y}$ the vector of all observed data.

Likelihood function The likelihood specifies the probability of the observed data given the parameters. We multiply the likelihoods corresponding to the three sub-models as introduced in the previous section. By assuming independence among dyads and trials (conditional upon the parameters), we have:

$$
\begin{aligned}
f(\mathbf{y}\,|\,\boldsymbol{\theta}) \;=\; & \prod_{i=1}^{10}\prod_{j=1}^{20} \Pr\left(XX_{ij} = x_{ij1}\cdot x_{ij2} \mid \beta_0^{(XX)}, \beta_D^{(XX)}\right) \\
& \times \prod_{i=1}^{10}\prod_{j=1}^{20} \Pr\left(X_{ij1} = x_{ij1} \text{ and } X_{ij2} = x_{ij2} \mid \boldsymbol{\tau}^{(X)}, \rho_{XX}\right) \\
& \times \prod_{i=1}^{10}\prod_{j=1}^{20} \Pr\left(Y_{ij1} = y_{ij1} \text{ and } Y_{ij2} = y_{ij2} \mid \boldsymbol{\tau}^{(Y)}, \rho_{YY}\right).
\end{aligned}
$$

The probabilities at the right hand side correspond with the probabilities for the data under the three sub-models and are obtained as specified in the respective subsections of Section 2. For convenience, we have omitted the dependency of the likelihood on the covariates included in Equations (S1), (S4), and (S8).

Prior distribution With respect to the prior distribution of the parameters:

- As explained before, the thresholds (in $\boldsymbol{\tau}^{(X)}$ and $\boldsymbol{\tau}^{(Y)}$) are fully determined by other parameters (see, Equations (S4) and (S8)) and, hence, we do not need to consider their (prior) distributions conditional upon the other parameters.
- The distributions of the random-effects parameters ($\beta_{0ik}^{(X)}$, $\beta_{1ik}^{(X)}$, $\zeta_{ik}^{(X)}$, $\beta_{0ik}^{(Y)}$, $\beta_{1ik}^{(Y)}$, and $\zeta_{ik}^{(Y)}$) are given in Equations (S5) and (S9).
- For the parameters $\mu_0^{(X)}$, $\sigma_0^{2(X)}$, $\mu_1^{(X)}$, and $\sigma_1^{2(X)}$ in Equations (S5) and the parameters $\mu_0^{(Y)}$, $\sigma_0^{2(Y)}$, $\mu_1^{(Y)}$, and $\sigma_1^{2(Y)}$ in Equations (S9), we specify the following vague prior distributions:

$$
\begin{aligned}
\mu_0^{(X)}, \mu_1^{(X)}, \mu_0^{(Y)}, \mu_1^{(Y)} &\overset{\text{iid}}{\sim} \text{Normal}(0, 10^2) \\
\sigma_0^{2(X)}, \sigma_1^{2(X)}, \sigma_0^{2(Y)}, \sigma_1^{2(Y)} &\overset{\text{iid}}{\sim} \text{Inv-Gamma}(0.1, 0.1)
\end{aligned}
$$

- With respect to the parameters $\alpha^{(X)}$ and $\beta^{(X)}$ of the beta distribution for the breakpoints $\zeta_{ik}^{(X)}$ (Eq. (S5c)) and, similarly, for $\alpha^{(X)}$ and $\beta^{(X)}$ of the beta distribution for the breakpoints $\zeta_{ik}^{(Y)}$ (Eq. (S9c)), we applied the following transformations in terms of parameters $\mu^{(X)}$, $\nu^{(X)}$, $\mu^{(Y)}$, $\nu^{(Y)}$:

$$
\begin{aligned}
\mu^{(X)} &= \frac{\alpha^{(X)}}{\alpha^{(X)} + \beta^{(X)}} & \nu^{(X)} &= \alpha^{(X)} + \beta^{(X)} \\
\mu^{(Y)} &= \frac{\alpha^{(Y)}}{\alpha^{(Y)} + \beta^{(Y)}} & \nu^{(Y)} &= \alpha^{(Y)} + \beta^{(Y)},
\end{aligned}
$$

  for which we specify the following priors:

$$
\mu^{(X)}, \mu^{(Y)} \overset{\text{iid}}{\sim} \text{Uniform}[0, 1] \qquad \nu^{(X)}, \nu^{(Y)} \overset{\text{iid}}{\sim} \text{Gamma}(1, 20).
$$

- The parameters δ_{12} and δ_{23} (corresponding to the distances between the thresholds; see (S7)) are assumed to be drawn from an exponential distribution:

$$
\delta_{12}, \delta_{23} \overset{\text{iid}}{\sim} \text{Expon}(1).
$$

Note that this distribution guarantees positive values for these parameters.

- For the regression parameters $\beta_0^{(XX)}$ and $\beta_D^{(XX)}$ (Eq. (S1)), $\beta_D^{(X)}$ (Eq. (S4)), and $\beta_X^{(Y)}$, $\beta_{XX}^{(Y)}$, and $\beta_D^{(Y)}$ (Eq. (S8)), a vague normal prior distribution is assumed:

$$\beta_0^{(XX)}, \beta_D^{(XX)}, \beta_D^{(X)}, \beta_X^{(Y)}, \beta_{XX}^{(Y)}, \beta_D^{(Y)} \overset{\text{iid}}{\sim} \text{Normal}(0, 10^2).$$

- The parameters ρ_{XX} and ρ_{YY} of the bivariate normal distributions for $(X_{ij1}^{\star}, X_{ij2}^{\star})$ (Eq. (S2)) and $(Y_{ij1}^{\star}, Y_{ij2}^{\star})$ (Eq. (S6)) are assumed to have the following uniform prior distribution:

$$\rho_{XX}, \rho_{YY} \overset{\text{iid}}{\sim} \text{Uniform}[-1, 1].$$

Sampling from the posterior distribution We obtained 5,000 draws from the posterior distribution by Markov chain Monte Carlo simulation; five chains, initialized at random starting points, were run for 600,000 iterations each, and the draw at every 600th iteration was saved. Convergence of the chains for each of the parameters was checked visually as well as by Gelman and Rubin's (1992) $\hat{R}$-statistic. The PROC MCMC procedure in SAS Software Version 9.4 (SAS Institute, 2015) was used for the analysis.

4 RESULTS AND INTERPRETATION

Table S1 summarizes the marginal posterior distribution for the parameters of principal interest. The posterior mean (which can be considered an estimate for the parameter), the posterior standard deviation (which quantifies the uncertainty for the estimate), and the 95%-high posterior density interval (which is a 95% credibility interval for the parameters) are presented. Note the relatively large posterior standard deviations and wide credibility intervals, implying a relatively high posterior uncertainty (due to the rather small sample size). This means that the results must be interpreted with care.

We now briefly discuss the results for each of the three sub-models.

4.1 Sub-model for joint success

With the estimates for $\beta_0^{(XX)}$ and $\beta_D^{(XX)}$, we calculated the model-based probabilities for joint success in case of a short inter-click delay (below 3 seconds) versus trials with no clicks or longer inter-click delays, which equal .74 and .36, respectively. With the 95%-credibility interval for the effect of short delays ($\beta_D^{(XX)}$) being large above zero, we can conclude that the results show strong evidence for this difference between these two types of trials.

4.2 Sub-model for individual success

As shown in Figure S1, an individual's successful recognition of the other player's avatar by producing a correct click during the trial is assumed to be affected by a learning process across trials and the dyad's inter-click delay. The individual's learning process was modeled as a piece-wise regression with first a learning stage, where the probability of recognizing the other increases, followed by a consolidation phase, where this probability stays the same (conditional upon the other effects in the model). The results show that the learning stage lasts from trial 1 to trial 3.2, *on average*. However, there is large variation (more precisely, a standard deviation of 2.9) across individuals with respect to this break point (i.e., the separation point between the learning and consolidation stages), as shown in Figure S2 (left panel). The latter figure is based on the parameter estimates for $\mu^{(X)}$ and $\nu^{(X)}$ (or, equivalently, $\alpha^{(X)}$ and $\beta^{(X)}$) and graphically represents the estimated population distribution for the break points. Indeed, a closer look at the data reveals large differences in the assumed learning process, with, for some individuals, high probabilities of

Table S1. Summary of the estimated marginal posterior distribution for the main parameters in the statistical model through the posterior mean and standard deviation, and the 95%-high posterior density interval.

Parameter	Posterior Mean	Posterior standard deviation	95%-high posterior density interval
$\beta_0^{(XX)}$	-0.35	0.10	$[-0.54; -0.16]$
$\beta_D^{(XX)}$	1.01	0.30	$[\ 0.40;\ 1.59]$
ρ_{XX}	0.52	0.12	$[\ 0.29;\ 0.74]$
$\mu_0^{(X)}$	0.18	0.13	$[-0.06;\ 0.43]$
$\sigma_0^{2(X)}$	0.15	0.09	$[\ 0.03;\ 0.33]$
$\mu_1^{(X)}$	0.07	2.05	$[-4.02;\ 4.71]$
$\sigma_1^{2(X)}$	29.35	29.62	$[\ 0.04;\ 88.50]$
$\mu^{(X)}$	0.12	0.05	$[\ 0.04;\ 0.21]$
$\nu^{(X)}$	4.91	5.41	$[\ 0.44;\ 13.78]$
$\alpha^{(X)}$	0.51	0.52	$[\ 0.07;\ 1.34]$
$\beta^{(X)}$	4.40	4.95	$[\ 0.26;\ 12.58]$
$\beta_D^{(X)}$	0.70	0.29	$[\ 0.16;\ 1.29]$
ρ_{YY}	0.04	0.14	$[-0.22;\ 0.31]$
δ_{12}	1.53	0.15	$[\ 1.23;\ 1.82]$
δ_{23}	1.13	0.11	$[\ 0.92;\ 1.34]$
$\mu_0^{(Y)}$	1.94	0.30	$[\ 1.36;\ 2.53]$
$\sigma_0^{2(Y)}$	0.57	0.28	$[\ 0.17;\ 1.12]$
$\mu_1^{(Y)}$	0.63	1.44	$[-0.01;\ 2.26]$
$\sigma_1^{2(Y)}$	0.58	5.36	$[\ 0.01;\ 1.06]$
$\mu^{(Y)}$	0.22	0.13	$[\ 0.00;\ 0.42]$
$\nu^{(Y)}$	19.73	14.26	$[\ 1.73;\ 50.93]$
$\alpha^{(Y)}$	4.03	4.08	$[\ 0.17;\ 12.41]$
$\beta^{(Y)}$	15.69	12.33	$[\ 0.94;\ 42.30]$
$\beta_X^{(Y)}$	-0.16	0.20	$[-0.55;\ 0.20]$
$\beta_{XX}^{(Y)}$	0.69	0.20	$[\ 0.31;\ 1.06]$
$\beta_D^{(Y)}$	0.14	0.21	$[-0.28;\ 0.54]$

recognizing the other from the start of the experiment (e.g., individual 1 of dyad 1, individual 1 of dyad 8, and individual 2 of dyad 9), while for others *in*correct clicks seem to be more common towards the end of the experiment (e.g., both individuals of dyad 4 and individual 2 from dyad 7) and for still other persons the pattern is unclear (e.g., individual 2 of dyad 3 and individual 2 of dyad 6). However, with X_{ijk} being a binary variable, with a sample of only 20 (interdependent) individuals, and under the assumption that the

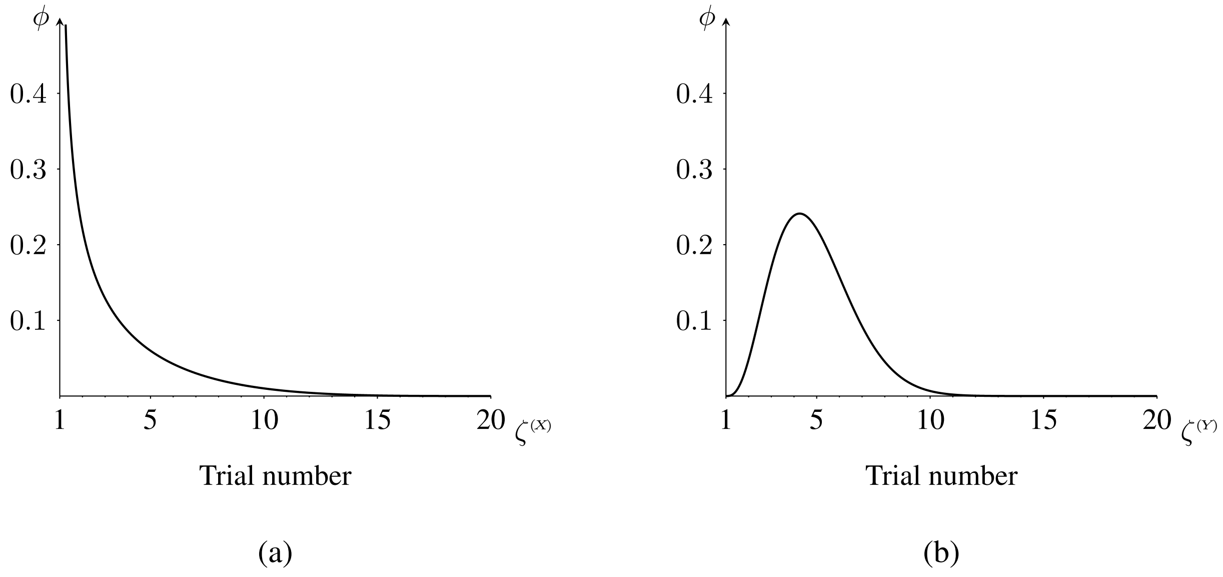

Figure S2. Estimated population distribution for (a) the individuals' break points $\zeta_{ik}^{(X)}$ (i.e., concerning the learning process assumed for correct recognition of the other) and (b) the individuals' break points $\zeta_{ik}^{(Y)}$ (i.e., concerning the learning process with regard to the perceptual awareness of the other's presence).

learning phase is limited to a (variable) number of trials at the beginning of the experiment, it should be clear that very little information is available to obtain robust findings in this respect. The main conclusion should probably be that our approach shows that individual differences are large and must be taken into account to reach correct conclusions. Note further that, as a consequence of this large variability and the particularly short duration of the (average) learning stage, the estimates associated with the increase during the learning stage (viz., $\mu_1^{(X)}$ and $\sigma_1^{2(X)}$) are highly uncertain and do not allow us to draw any conclusions.

The average level of individual success (reached towards the end of the experiment, i.e., during the consolidation stage) is quantified by the parameter $\mu_0^{(X)}$. The estimated value of 0.18 for this parameter corresponds with a probability of success equal to .57; this probability applies to the case of a trial where the inter-click delay is above 3 seconds (or the other player did not click). If the inter-click delay is below 3 seconds, this probability increases to .81. Again, we observe a strong and reliable effect of short inter-click delays.

Finally, an interesting finding is that the correlation ρ_{XX} between both players in a dyad with respect to their (latent) abilities of recognizing the other is estimated at .52. A correct interpretation of this correlation implies considering all other effects in the model constant, that is, the correlation that remains after taking into account the learning process and the possible effect of short inter-click delays.

4.3 Sub-model for the PAS-response

Our model was specified assuming that the perceptual awareness that an individual has of the other player during a trial is influenced by four different effects: a learning process, individual and joint success in recognizing the other (i.e., the click response), and the inter-click delay.

Again, the learning process was supposed to consist of a learning stage and a consolidation stage. The average break point (separating the learning and consolidation stages) was estimated to be at trial 5.1 with an estimated standard deviation of 1.9. More precisely, the estimated population distribution of these break

points is graphically depicted in Figure S2 (right panel). In spite of the still high variability, the standard deviation is substantially smaller in this case as compared to the case discussed in the previous subsection (see the left panel of the same figure) and the results are easier to interpret. The estimates for $\mu_1^{(Y)}$ and $\sigma_1^{2(Y)}$ imply that, during the learning stage, the average increase is 0.63 units (on the latent probit scale) per trial, with a rather large variance of 0.58. This implies that most people first go through a learning stage where their perceptual awareness of the other gradually increases; looking at the random effects $\beta_{1ik}^{(Y)}$ shows that the point estimates for these parameters are positive for all the 20 individuals in our sample (although accompanied by large credibility intervals that in almost all cases include negative values as well).

The parameter $\mu_0^{(Y)}$ indicates the average level of perceptual awareness reached after the learning stage; the estimate of 1.94 for this parameter, together with the estimates for δ_{12} and δ_{23}, allows us to calculate the average model-based probability of an individual responding in any of the four categories of the PAS-question. For trials where the person did not produce a correct click and the dyad's inter-click delay was not below 3 seconds, these probabilities are .03, .32, .42, and .23 for categories 0 ("having had no experience of the other"), 1 ("having had an ambiguous experience of the other"), 2 ("having had an almost clear experience of the other") and 3 ("having had a clear experience of the other"), respectively.

The parameters $\beta_X^{(Y)}$ and $\beta_{XX}^{(Y)}$ quantify how individual and/or joint success in recognizing the other (through correct clicking) modifies the latter probabilities. The estimate for $\beta_X^{(Y)}$ is close to zero and, hence, an individual having correctly recognized the other player does not affect her reported perceptual awareness of the other. Indeed, the probabilities of responding in the four PAS-categories reported in the previous paragraph hardly change to .04, .37, .41, and .19, respectively. However, *mutual* recognition has a strong effect on perceptual awareness. For trials where both individuals correctly recognized each other, the probabilities to respond in the four PAS-categories change to .01, .17, .40, and .42, respectively. In other words, the probability to report "having had a clear experience of the other" almost doubles when both players mutually recognized each other.

There is no evidence that the short inter-click delays are directly related with responses in the higher categories of the PAS-question; although the estimate for $\beta_X^{(Y)}$ is slightly positive (0.14), the associated credibility interval does not exclude negative values. However, as shown previously, an indirect effect may hold, given that, on the one hand, short-click delays are associated with higher probabilities of (mutual and individual) successful recognition of the other and, on the other hand, mutual successful recognition of the other is associated with higher probabilities of a clear perceptual awareness.

Finally, the residual correlation ρ_{YY} between the (latent) perceptual awareness of both individuals in a dyad is close to zero. This does not mean that there is no association between the perceptual awareness of both individuals, but rather that the other effects included in the model (the learning process, individual and joint success in recognizing the other, and the inter-click delay) may account for this correlation.

REFERENCES

Rubin DB. Inference and missing data. *Biometrika* **63** (1976) 581–592. doi:10.2307/2335739.

Gelman A, Carlin JB, Stern HS, Dunson DB, Vehtari A, Rubin DB. *Bayesian Data Analysis* (Boca Raton, FL: Chapman & Hall/CRC), 3rd edn. (2014).

Gelman A, Rubin DB. Inference from iterative simulation using multiple sequences. *Statistical Science* **7** (1992) 457–511. doi:10.1214/ss/1177011136.

SAS Institute. *SAS/STAT® 14.1 User's Guide* (Cary, NC: Author) (2015).

APPENDIX

The following table shows the data to which the statistical model was fitted. The same data are also available as a separate file in csv-format:

Dyad index	Trial index	PAS-response		Individual success		Joint success	Inter-click delay	
i	j	Y_{ij1}	Y_{ij2}	X_{ij1}	X_{ij2}	XX_{ij}	seconds	D_{ij}
1	1	2	.	1	0	0	.	0
1	2	2	.	1	0	0	.	0
1	3	2	.	1	0	0	.	0
1	4	3	2	1	1	1	27.555	0
1	5	3	3	1	1	1	4.857	0
1	6	3	3	1	1	1	0.560	1
1	7	3	3	1	1	1	9.336	0
1	8	4	4	1	1	1	36.780	0
1	9	4	4	1	1	1	34.517	0
1	10	2	.	1	0	0	.	0
1	11	4	4	1	1	1	3.933	0
1	12	4	4	1	1	1	11.030	0
1	13	4	4	1	1	1	3.937	0
1	14	4	4	1	1	1	13.109	0
1	15	4	4	1	1	1	14.229	0
2	1	1	.	0	0	0	.	0
2	2	2	3	0	0	0	29.249	0
2	3	3	2	1	1	1	51.766	0
2	4	3	2	0	1	0	37.410	0
2	5	2	.	0	0	0	.	0
2	6	3	2	0	0	0	3.944	0
2	7	4	3	0	0	0	30.415	0
2	8	3	.	0	0	0	.	0
2	9	4	3	1	1	1	17.399	0
2	10	.	1	0	1	0	.	0
2	11	.	.	0	0	0	.	0
2	12	3	3	1	1	1	11.185	0
2	13	4	3	1	0	0	34.299	0
2	14	.	.	0	0	0	.	0
2	15	.	.	0	1	0	.	0
2	16	4	3	1	1	1	7.371	0
2	17	4	3	1	1	1	15.048	0
2	18	4	3	1	1	1	14.537	0
2	19	4	3	1	1	1	14.554	0
2	20	.	3	0	0	0	.	0
3	1	2	2	0	0	0	11.293	0
3	2	2	2	0	0	0	26.262	0
3	3	.	2	0	1	0	.	0

Dyad index	Trial index	PAS-response		Individual success		Joint success	Inter-click delay	
i	j	Y_{ij1}	Y_{ij2}	X_{ij1}	X_{ij2}	XX_{ij}	seconds	D_{ij}
3	4	2	.	0	0	0	.	0
3	5	1	.	0	0	0	.	0
3	6	2	3	1	0	0	14.922	0
3	7	3	4	1	1	1	7.525	0
3	8	2	2	0	1	0	24.932	0
3	9	3	4	1	1	1	20.089	0
3	10	2	2	0	1	0	38.184	0
3	11	3	2	0	1	0	9.022	0
3	12	4	3	0	0	0	7.582	0
3	13	3	4	1	0	0	27.434	0
3	14	4	4	1	1	1	8.653	0
3	15	4	2	0	0	0	3.958	0
3	16	4	2	1	1	1	28.555	0
3	17	2	4	1	1	1	3.297	0
3	18	2	.	1	0	0	.	0
3	19	3	3	0	0	0	2.837	1
3	20	4	3	0	0	0	1.365	1
4	1	2	3	1	1	1	45.710	0
4	2	2	2	1	1	1	16.013	0
4	3	.	3	0	1	0	.	0
4	4	3	2	0	0	0	0.041	1
4	5	3	1	1	1	1	6.686	0
4	6	4	.	0	0	0	.	0
4	7	3	4	1	1	1	11.170	0
4	8	.	4	0	1	0	.	0
4	9	.	4	0	1	0	.	0
4	10	3	3	0	1	0	20.087	0
4	11	4	2	1	1	1	5.583	0
4	12	.	2	0	1	0	.	0
4	13	4	2	1	1	1	16.416	0
4	14	4	3	1	1	1	9.000	0
4	15	.	2	0	1	0	.	0
4	16	2	.	0	0	0	.	0
4	17	.	.	0	0	0	.	0
4	18	3	2	1	1	1	2.735	1
4	19	.	.	0	0	0	.	0
4	20	.	2	0	0	0	.	0
5	1	2	4	0	1	0	41.787	0
5	2	.	4	0	1	0	.	0
5	3	.	2	0	0	0	.	0
5	4	2	2	1	0	0	15.387	0
5	5	3	3	1	0	0	11.985	0

Dyad index	Trial index	PAS-response		Individual success		Joint success	Inter-click delay	
i	j	Y_{ij1}	Y_{ij2}	X_{ij1}	X_{ij2}	XX_{ij}	seconds	D_{ij}
5	6	4	4	1	1	1	3.415	0
5	7	3	4	0	1	0	14.015	0
5	8	.	3	0	1	0	.	0
5	9	.	4	0	0	0	.	0
5	10	2	4	1	1	1	6.335	0
5	11	.	2	0	1	0	.	0
5	12	4	4	1	1	1	6.872	0
5	13	4	4	1	1	1	5.670	0
5	14	4	4	1	1	1	1.562	1
5	15	4	4	1	1	1	1.202	1
5	16	4	4	1	1	1	3.697	0
5	17	4	4	1	1	1	14.192	0
5	18	4	3	1	1	1	0.948	1
5	19	4	3	1	1	1	3.742	0
5	20	4	4	1	0	0	5.455	0
6	1	3	1	0	1	0	47.222	0
6	2	4	2	1	1	1	17.396	0
6	3	4	2	0	0	0	31.315	0
6	4	4	3	0	0	0	41.798	0
6	5	4	2	1	1	1	13.890	0
6	6	.	2	0	0	0	.	0
6	7	4	3	1	1	1	0.944	1
6	8	4	2	0	1	0	7.716	0
6	9	2	2	1	1	1	4.595	0
6	10	.	3	0	0	0	.	0
6	11	4	3	1	1	1	28.909	0
6	12	3	1	1	1	1	10.130	0
6	13	.	.	0	1	0	17.017	0
6	14	3	3	1	0	0	2.878	1
6	15	4	.	1	0	0	.	0
6	16	4	3	1	1	1	3.347	0
6	17	.	.	0	0	0	.	0
6	18	4	3	1	1	1	16.540	0
6	19	.	3	1	1	1	12.127	0
7	1	3	1	1	1	1	7.329	0
7	2	2	2	0	1	0	5.096	0
7	3	2	2	0	1	0	6.264	0
7	4	2	2	1	1	1	1.946	1
7	5	3	2	0	1	0	13.972	0
7	6	3	1	0	1	0	4.371	0
7	7	3	3	1	1	1	0.030	1
7	8	2	.	0	0	0	.	0

Dyad index	Trial index	PAS-response		Individual success		Joint success	Inter-click delay	
i	j	Y_{ij1}	Y_{ij2}	X_{ij1}	X_{ij2}	XX_{ij}	seconds	D_{ij}
7	9	2	.	1	0	0	.	0
7	10	3	3	1	1	1	17.152	0
7	11	3	3	1	0	0	29.395	0
7	12	2	.	1	0	0	.	0
7	13	3	.	1	0	0	.	0
7	14	3	.	1	0	0	.	0
7	15	4	3	1	1	1	16.737	0
7	16	2	3	1	1	1	15.405	0
7	17	1	.	1	0	0	.	0
7	18	2	3	1	1	1	6.241	0
7	19	2	.	1	0	0	.	0
7	20	2	3	1	1	1	24.774	0
8	1	4	2	1	1	1	12.513	0
8	2	3	3	1	0	0	37.317	0
8	3	3	.	1	0	0	.	0
8	4	4	4	1	1	1	5.192	0
8	5	.	2	0	0	0	.	0
8	6	4	2	1	1	1	2.481	1
8	7	4	4	1	1	1	0.556	1
8	8	4	.	1	0	0	.	0
8	9	2	.	1	0	0	.	0
8	10	4	4	1	1	1	0.692	1
8	11	.	2	0	1	0	.	0
8	12	2	3	0	0	0	7.627	0
8	13	4	3	1	1	1	2.932	1
8	14	3	4	1	1	1	0.481	1
8	15	4	3	1	1	1	3.796	0
8	16	4	4	1	1	1	1.356	1
8	17	.	.	0	0	0	.	0
8	18	4	2	1	1	1	0.144	1
8	19	3	3	1	1	1	0.989	1
8	20	2	3	0	0	0	3.006	0
9	1	2	2	1	1	1	1.775	1
9	2	1	2	1	1	1	3.362	0
9	3	2	3	0	1	0	27.628	0
9	4	2	.	1	0	0	.	0
9	5	3	2	0	1	0	32.404	0
9	6	1	3	0	1	0	4.663	0
9	7	2	.	0	0	0	.	0
9	8	2	2	1	1	1	3.630	0
9	9	2	2	1	0	0	30.364	0
9	10	3	3	1	1	1	34.280	0

Dyad index	Trial index	PAS-response		Individual success		Joint success	Inter-click delay	
i	j	Y_{ij1}	Y_{ij2}	X_{ij1}	X_{ij2}	XX_{ij}	seconds	D_{ij}
9	11	.	4	0	1	0	.	0
9	12	.	.	0	0	0	.	0
9	13	1	3	0	1	0	30.704	0
9	14	1	4	1	1	1	15.463	0
9	15	3	3	1	1	1	29.261	0
9	16	2	3	1	1	1	8.350	0
9	17	1	3	1	1	1	3.264	0
9	18	1	2	1	1	1	5.822	0
9	19	1	4	1	1	1	3.459	0
9	20	.	4	0	0	0	.	0
10	1	3	2	0	0	0	1.372	1
10	2	.	.	0	0	0	.	0
10	3	2	3	1	0	0	17.311	0
10	4	1	.	1	0	0	.	0
10	5	4	.	1	0	0	.	0
10	6	.	1	0	0	0	.	0
10	7	3	2	1	0	0	32.370	0
10	8	3	.	0	0	0	.	0
10	9	2	.	0	0	0	.	0
10	10	3	.	0	0	0	.	0
10	11	3	.	1	0	0	.	0
10	12	4	1	0	0	0	0.334	1
10	13	.	2	0	1	0	.	0
10	14	.	4	0	1	0	.	0
10	15	4	.	0	0	0	.	0
10	16	3	4	0	1	0	4.298	0
10	17	.	.	0	0	0	.	0
10	18	.	2	0	1	0	.	0
10	19	.	.	0	0	0	.	0
10	20	3	1	0	1	0	33.082	0

Finally, the following table provides some details on the participants:

Dyad index i	*Strangers**	Player 1		Player 2	
		Sex	*Age*	*Sex*	*Age*
1	No	Male	47	Male	40
2	No	Male	23	Male	18
3	Yes	Male	36	Female	40
4	No	Female	23	Female	23
5	No	Female	37	Male	21
6	Yes	Female	43	Male	44
7	No	Male	28	Male	27
8	No	Male	27	Male	28
9	No	Male	25	Male	24
10	Yes	Female	35	Male	26

*This column indicates whether the members of the dyad knew each other or not (*Strangers* = No and *Strangers* = Yes, resp.).
